# Impact of intensified control on visceral leishmaniasis in a highly-endemic district of Bihar, India: an interrupted time series analysis

**DOI:** 10.1016/j.epidem.2022.100562

**Published:** 2022-06

**Authors:** Vijay Kumar, Niyamat A. Siddiqui, Timothy M. Pollington, Rakesh Mandal, Sushmita Das, Shreekant Kesari, Vidyanand R. Das, Krishna Pandey, T. Déirdre Hollingsworth, Lloyd A.C. Chapman, Pradeep Das

**Affiliations:** a Rajendra Memorial Research Institute of Medical Sciences (RMRIMS) (ICMR), Patna 800007, India; b MathSys - Mathematics for Real-World Systems Centre for Doctoral Training, University of Warwick, Coventry CV4 7AL, UK; c Big Data Institute (BDI), University of Oxford, Oxford OX3 7LF, UK; d Centre for Tropical Medicine and Global Health, University of Oxford, Oxford OX3 7LG, UK; e Mahidol-Oxford Tropical Medicine Research Unit (MORU), Mahidol University, Bangkok 10400, Thailand; f All India Institute of Medical Sciences (AIIMS), Patna 801507, India; g Centre for Mathematical Modelling of Infectious Diseases (CMMID), London School of Hygiene & Tropical Medicine (LSHTM), London WC1H 9SH, UK; h National Institute of Cholera and Enteric Diseases (NICED) (ICMR), Kolkata 700010, India

**Keywords:** *ACD*, active case detection, *ASHA*s, accredited social health activists, *DD*, diagnosis-to-diagnosis, *IEC*, information, education & communication, *ITSA*, interrupted time series analysis, *PCD*, passive case detection, *PIT*, probability integral transform, *OD*, onset-to-diagnosis, Kala-azar, Integrated control, Distributed-lag, Regression discontinuity, Spatiotemporal, Elimination

## Abstract

Visceral leishmaniasis (VL) is declining in India and the World Health Organization’s (WHO) 2020 ‘elimination as a public health problem’ target has nearly been achieved. Intensified combined interventions might help reach elimination, but their impact has not been assessed. WHO’s Neglected Tropical Diseases 2021–2030 roadmap provides an opportunity to revisit VL control strategies. We estimated the combined effect of a district-wide pilot of intensified interventions in the highly-endemic Vaishali district, where cases fell from 3,598 in 2012–2014 to 762 in 2015–2017. The intensified control approach comprised indoor residual spraying with improved supervision; VL-specific training for accredited social health activists to reduce onset-to-diagnosis time; and increased Information Education & Communication activities in the community. We compared the rate of incidence decrease in Vaishali to other districts in Bihar state via an interrupted time series analysis with a spatiotemporal model informed by previous VL epidemiological estimates. Changes in Vaishali’s rank among Bihar’s endemic districts in terms of monthly incidence showed a change pre-pilot (3rd highest out of 33 reporting districts) vs. during the pilot (9th) (p<1e-10). The rate of decline in Vaishali’s incidence saw no change in rank at 11th highest, both pre-pilot & during the pilot. Counterfactual model simulations suggest an estimated median of 352 cases (IQR 234–477) were averted by the Vaishali pilot between January 2015 and December 2017, which was robust to modest changes in the onset-to-diagnosis distribution. Strengthening control strategies may have precipitated a substantial change in VL incidence in Vaishali and suggests this approach should be piloted in other highly-endemic districts.

## Graphical abstract

**Figure:**
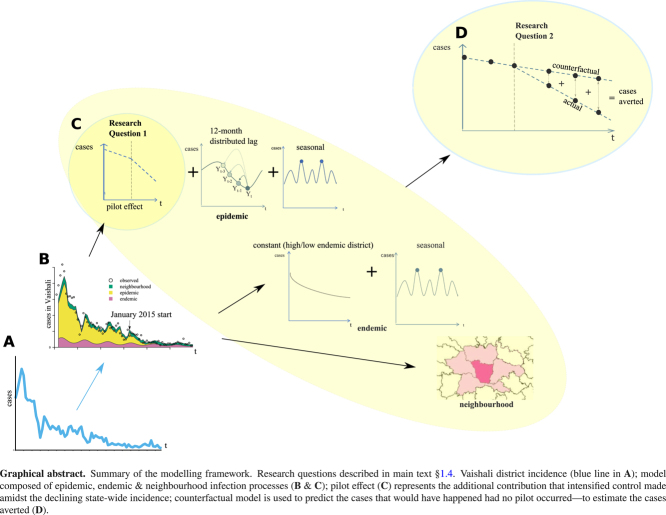

## 1 Introduction

The infectious disease *visceral leishmaniasis* (VL) still persists in India despite large-scale elimination efforts. The clinical form of the disease is usually fatal without treatment. India had an estimated 146,700–282,800 VL cases annually between 2004–2008, most of which were from Bihar state (Alvar et al., 2012). VL cases have declined since 2011 but have plateaued slightly in recent years (WHO, 2017, NVBDCP, 2018). The World Health Organization’s (WHO) 2020 target for elimination of VL as a public health problem (<1 case/10,000 people/year at block (subdistrict) level) (WHO, 2012, Hirve et al., 2017) has now passed and only ∼2% of blocks are still above the target (February 2021), but resurgence may still occur—as seen in previous decadal cycles (Dye and Wolpert, 1988). Therefore, analysis from this recent intensified pilot is still relevant for future control policy.

### 1.1 Routine VL control in Bihar state

Current interventions implemented by the National Vector Borne Disease Control Programme (NVBDCP) involve biannual indoor residual spraying (IRS) of insecticide at state-level, passive case detection (PCD) at (block-level) by primary health centres and active case detection (ACD) by accredited social health activists (ASHAs); or via annual mobile camps (*Supplementary information* (SI) §S1).

### 1.2 Existing research base for interventions

An IRS review in Bangladesh, Nepal & India showed IRS had an impact on sandfly densities when properly conducted but did not show a significant impact on VL case incidence (Picado et al., 2012). The only large-scale randomised control trial of a vector control intervention on infection incidence (the KALANET project) found no evidence that large-scale distribution of long-lasting insecticidal nets provided additional protection over existing control practices (Picado et al., 2010, Picado et al., 2015). A multi-site ACD screening intervention by ASHAs in highly-endemic Muzaffarpur & Saran districts discovered 6·7–17·1% more cases than PCD alone (Hirve et al., 2010). Overall, robust evidence is lacking on intervention effectiveness from field trials. Nevertheless, we hypothesise that a *combination* of strengthening the ACD referral system through VL-specific training for ASHAs, higher quality IRS by well-trained & supervised spray teams, and information, education & communication (IEC) community activities (SI §S2) could produce measurable incidence reductions. It is expensive to run a control programme of this scale: requiring coordination between Rajendra Memorial Research Institute of Medical Sciences (RMRIMS) and the Ministry of Health & Family Welfare, for administrative & logistical support and needing 166 spray squads for several weeks, twice a year (SI §S2). Therefore, any policy decision to apply this costly intervention to highly-endemic districts in future requires an evidence base.

**Fig. 1:**
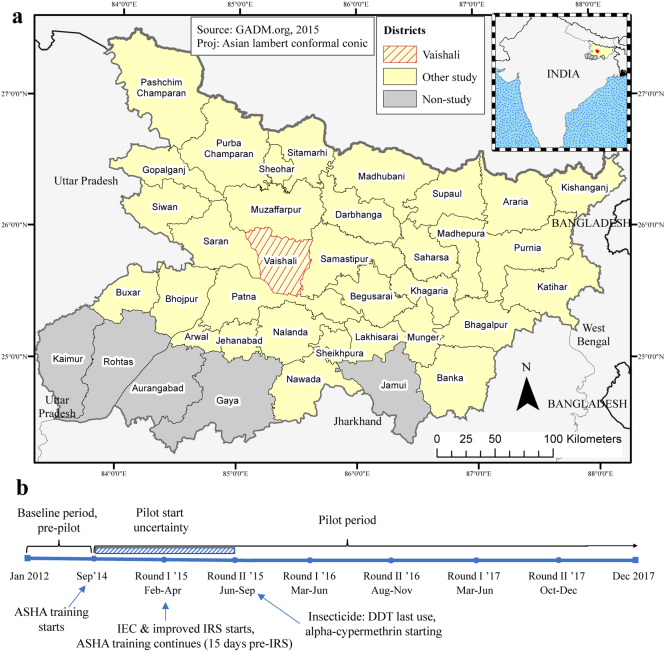
Study map & timeline. (a) The pilot district of Vaishali is the hatched region. GADM shapefile (GADM, 2015). (b) Annotations indicate the start months of the intensified control elements and circular dots mark the biannual accredited social health activist (ASHA) training, indoor residual spraying (IRS) training rounds, and information, education & communication activities (IEC). The hatched bar marks the period of pilot scale-up when the combined methods would unlikely have reached full impact. Made in ArcMap™.

### 1.3 Intensified control in Vaishali district

RMRIMS conducted an observational study on the impact of intensified VL control covering 1,569 villages in all 16 blocks in Vaishali district in late 2014–early 2015 (when 15 blocks were above the elimination threshold), while standard control by the NVBDCP continued in other districts (Fig. 1 & SI §S1–2) (Kumar et al., 2020). The triad of ongoing interventions, which began asynchronously, are specialised ASHA training (21–29 September 2014), improved IRS (from 15 February 2015) & IEC (19–21 February 2015) (Fig. 1b & SI §S2).

### 1.4 Research questions & rationale for spatiotemporal model

**Fig. 2:**
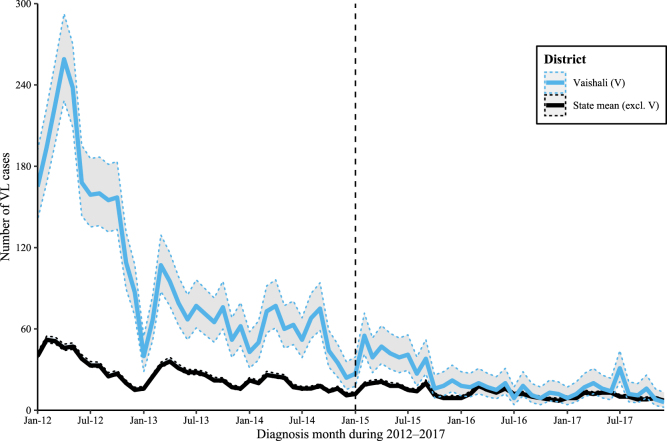
Visceral leishmaniasis time series for Vaishali district and the rest of Bihar state. Monthly case counts (Government of India, 2017). Note that HIV-VL cases are included from 2015–2016 and HIV/TB-VL from 2017; monthly VL-HIV/TB case proportions are shown in SI Fig. S9. The state mean excludes Aurangabad, Gaya, Jamui, Kaimur, Rohtas & Vaishali districts. The dashed vertical line indicates the start of the modelled intervention.

In this study we estimate: (i) *whether intensified control additionally contributed to the decline in VL cases in Vaishali, versus other districts* (RQ1), & (ii) *how many VL cases were averted by the pilot?* (RQ2). Answering these questions is complicated since incidence was already falling in Vaishali before the pilot started (Fig. 2). Crude calculations indicate decreasing case counts year-on-year: 664 in 2014, falling by 38·1% to 411 in 2015, and by 56·4% to 179 in 2016 (Kumar et al., 2017). To estimate the impact of the pilot while accounting for the decreasing background secular trend, we compared Vaishali with other districts rather than analysing it in isolation. The model is informed by prior VL epidemiology & spatiotemporal features of the setting (SI §S6–7) (Bern et al., 2010). To estimate the number of cases averted, we fit the same model to a subset of pre-intervention months and make counterfactual predictions of case counts with which observed case counts can be compared (Graphical abstract & SI §S11).

## 2 Methods

### 2.1 Longitudinal dataset

Monthly VL case counts (by diagnosis date) for 33 out of the 38 districts of Bihar from January 2012–December 2017 were provided by the State Vector Borne Disease Office (Government of India, 2017). Our analysis included HIV-VL cases from January 2015–December 2016 and HIV/TB-VL cases from January 2017–December 2017 but excluded post–kala-azar dermal leishmaniasis (PKDL) cases (SI Fig. S9). The 33 study districts formed a contiguous island of transmission without the five remaining districts (Aurangabad, Gaya, Jamui, Kaimur & Rohtas, which are considered non-endemic) (Fig. 1a). Monthly district populations were estimated from 2001 & 2011 censuses (Government of India, 2015). District shapefiles provided adjacency information (GADM, 2015). The Institutional Ethical Committee of RMRIMS approved the intensified control programme (03/RMRI/EC/2018). University of Warwick’s Biomedical & Scientific Research Ethics Committee (REGO-2018-2231) approved this analysis.

### 2.2 Descriptive analysis

Districts were compared by their ranked incidence levels and year-on-year changes in monthly incidence (SI §S3). Changes in rank position enabled us to crudely compare the *relative* changes of Vaishali to other districts, in the context of a state-wide medium-term decline in incidence. Using the two-sample two-tailed Wilcoxon test with continuity correction, we assessed if the ranks before & during the pilot were different. Evidence for global spatial correlation in incidence was assessed before & during the pilot with a Global Moran’s I statistic hypothesis test (SI §S4). The effective reproduction number ${\overset{ˆ}{R}}_{e}\left(t\right)$ for Vaishali & non-pilot districts was estimated to explore temporal patterns in transmission that may have been affected by interventions or seasonality (SI §S5) (Cori et al., 2013, Cori et al., 2020).

### 2.3 Interrupted time series analysis (ITSA)

*ITSA* is a subset of regression discontinuity analysis which we applied to districts’ longitudinal case counts to assess the impact of this non-randomised pilot while adjusting for existing trends (Kontopantelis et al., 2015, Bärnighausen et al., 2017, Bernal et al., 2017). The *assignment variable* in this ITSA is the calendar time τ (Eqn. 2:2) of the start of the pilot implementation in Vaishali. We evaluated the dynamics of case counts before & during the pilot using a spatiotemporal framework (Held et al., 2005, Held et al., 2017, Meyer et al., 2017, Bracher and Held, 2020, R, 2020).

The process producing observed cases $Y_{i,t}$ in any district i in diagnosis month t is assumed to follow a Negative Binomial distribution with mean $\mu_{i,t}$ & variance $\sigma_{i,t}^{2}$ conditional on a weighted sum of cases from the previous 12 months $\sum_{T=1}^{12}D_{T}Y_{i,t-T}$, where $D_{T}$ is the weight for the cases T months ago (*i.e.* *distributed-lag* autoregression) (Eqn. 1 & SI §S7). This distributed-lag distribution represents the *diagnosis-to-diagnosis* (DD) distribution, *i.e.* the distribution of times between VL *diagnoses* of infector & infectee (SI §S6), akin to a ‘diagnosis’ serial interval distribution. It better represents the temporal correlation of diagnosis times than a naïve lag-1 autoregression (Bracher and Held, 2020). The normalised DD distribution $D_{T}$ is informed by an estimated incubation period (mean = 6 mo) (Chapman et al., 2015, Chapman et al., 2020), which broadly agrees with literature estimates (Boelaert and Sundar, 2014), and an onset-to-diagnosis (OD) distribution (mean = 1·47 mo) from a Bihar study in the third quarter of 2012 (Jervis et al., 2017). With the DD distribution being a central assumption of all our models, we focussed a sensitivity analysis on the OD distribution to assess impact on RQ1 & RQ2 (SI §S12).

The base model of monthly district case counts (Eqn. 1) represents ongoing direct transmission between cases while accounting for the typical VL DD interval (‘epidemic’ component, λ), hidden transmission from unobserved or asymptomatic cases (‘endemic’ component, ν) with high/low-incidence stratification $\alpha_{i,t}^{\left(\nu \right)}\in \left\{\alpha_{\text{low incid.}}^{\left(\nu \right)},\alpha_{\text{high incid.}}^{\left(\nu \right)}\right\}$, effects of directly-adjacent districts (‘neighbourhood’ component, ϕ) (SI §S7–8), and changing district-specific population effects (SI Fig. S3 & S5).

(1) $$Y_{i,t}|\left\{Y_{i,t-12},\ldots ,Y_{i,t-1}\right\}∼\text{NegBin}\left(\mu_{i,t},\sigma_{i,t}^{2}\right),\mu_{i,t}=e_{i,t}\nu_{i,t}+\lambda_{i}\overset{12}{\underset{T=1}{\sum }}D_{T}Y_{i,t-T}+\phi_{i}\underset{j\neq i}{\sum }\left(\omega_{ji}\overset{12}{\underset{T=1}{\sum }}D_{T}Y_{i,t-T}\right)$$

with population offset $e_{i,t}$; $\omega_{ji}=1$ if j neighbours i, else 0; and two overdispersion terms $\psi_{\text{high}},\psi_{\text{low}}>0$, *s.t.* $\sigma_{i,t}^{2}=\mu_{i,t}\left(1+\psi_{k}\mu_{i,t}\right)$ for k∈{high,low} endemicity districts (SI §S8).

We expanded the base model with the pilot effect (*apriori* primary variable, $\alpha_{\text{pilot}}^{\left(\lambda \right)})$ and annual seasonality in the epidemic & endemic components (Eqns. 2:1 & 2:2, SI §S9) to form the final model:

$$\ln\left(\nu_{i,t}\right)=\alpha_{i,t}^{\left(\nu \right)}+A_{\text{END}}\sin\left(\frac{2\pi }{12}t+\Phi_{\text{END}}\right)\;\;\;\;\text{(endemic, 2:1)}\ln\left(\lambda_{i,t}\right)=\alpha_{\text{other}}^{\left(\lambda \right)}+1_{\left\{i=\text{Vaishali}\right\}}\left(\alpha_{\text{Vaishali}}^{\left(\lambda \right)}+1_{\left\{t\geq \tau \right\}}c_{t}\cdot \alpha_{\text{pilot}}^{\left(\lambda \right)}\right)\;\;\;+A_{\text{AR}}\sin\left(\frac{2\pi }{12}t+\Phi_{\text{AR}}\right)\;\;\;\;\text{(epidemic, 2:2)}\ln\left(\phi_{i}\right)=\alpha_{i}^{\left(\phi \right)}\;\;\;\;\;\;\;\;\;\;\;\text{(neighbourhood, 2:3)}$$

with $A_{\left(\text{END/AR}\right)}$ annual sinusoid amplitude & phase $\Phi_{\left(\text{END/AR}\right)}$, fixed intercept means for the 32 districts $\alpha_{\text{other}}^{\left(\lambda \right)}$, and Vaishali $\left(\alpha_{\text{other}}^{\left(\lambda \right)}+\alpha_{\text{Vaishali}}^{\left(\lambda \right)}\right)$, and corrections $c_{t}$ for the first 12 months of the pilot due to delayed-lag intervention effects (SI §S9).

Model selection was based on the lowest Akaike information criterion (AIC) of each candidate model versus the best-performing model from the previous selection step, while monitoring changes in parameter uncertainty, particularly for the primary variable (Paul and Held, 2011). A range of possible pilot start months τ were tested (September 2014–September 2015 inclusive), and the most likely month chosen based on AIC. To assess the sensitivity of the final model’s parameters to τ, we also reported their range when the start month was varied.

For model validation (SI §S10) predictive performance in the pre-pilot period was visually assessed through fanplots & probability integral transform (PIT) histograms (Czado et al., 2009, Paul and Held, 2011, Abel, 2015, Meyer et al., 2017). The fitted values of the final model’s case counts were plotted against their residuals to assess heteroskedasticity.

### 2.4 Counterfactual model

A *counterfactual model*, formed by omitting from Eqn. 2:2 the $\left(1_{\left\{t\geq \tau \right\}}c_{t}\cdot \alpha_{\text{pilot}}^{\left(\lambda \right)}\right)$ term, was used to predict the number of cases that would have occurred had there been no intensified control in Vaishali. Informed by the most likely start month $\tau^{*}$, we estimated the cases averted in Vaishali during January 2015–December 2017 by summing the monthly differences between simulated case counts from this counterfactual (fitted on January 2013–December 2014), and the final model (SI §S11). Cases averted were also presented as a percentage of those that would have occurred under the counterfactual model (Graphical abstract:D).

When developing a single model to both infer the pilot effect (RQ1) & generate counterfactual predictions (RQ2), it is unclear how to weight AIC & predictive performance (SI §S10) for these respective purposes (Nightingale et al., 2020). So pragmatically, we optimised for fit first; and prediction second, working from the pilot model. For model validation, the base, pilot & counterfactual models’ goodness of fit were compared by AIC for the period January 2013–December 2017 (SI §S10). The predictive performance of the base & final models for January 2015–November 2017 was also compared (SI §S9).

## 3 Results

### 3.1 Trends in diagnoses

Case counts fell in most districts, including Vaishali, from 2012–2017 (Fig. 2). During the pilot years 2015–2017, monthly cases in Vaishali declined substantially in absolute terms compared with cases in its highly-endemic neighbours and the district mean of the rest of the state (Figs. 1a & 2).

Both during & before the pilot, Vaishali had the 11th largest year-on-year percentage reduction in monthly VL incidence out of 33 reporting districts (averaged over 36 monthly incidence ranks from 2015–2017 and over 24 months from 2013–2014 respectively). It also had the 9th highest VL incidence during the pilot period (averaged over 36 monthly incidence ranks from 2015–2017) versus pre-pilot when it was the 3${}^{\text{rd}}$ highest (averaged over 36 months from 2012–2014, p<1e-10 Wilcoxon test).

### 3.2 Seasonality & spatial correlation

Across Bihar, an annual seasonality in case counts was apparent, whose signal weakened as endemicity fell (Fig. 2). However, at district-level the strength of the seasonal signal varied and was only recognisable for some high-endemicity districts (*e.g.* Saran had a strong seasonal signal while others did not—note not presented in figures.).

Spatial correlation in incidence between neighbouring districts was apparent both before & during the pilot; Global Moran’s I=0·36, p=0·002 (10,000 simulations) and I=0·40, p=8e-04, respectively. This supports the use of the between-district neighbourhood component in Eqn. 2:3. Vaishali was surrounded by neighbours with a range of endemicities, which either remained constant (*e.g.* Saran) or declined. Although incidence was declining in Vaishali, it was also declining in many other districts, yet clustering remained among the other districts, while Vaishali was dissimilar to its neighbours.

### 3.3 Effective reproduction number

**Fig. 3:**
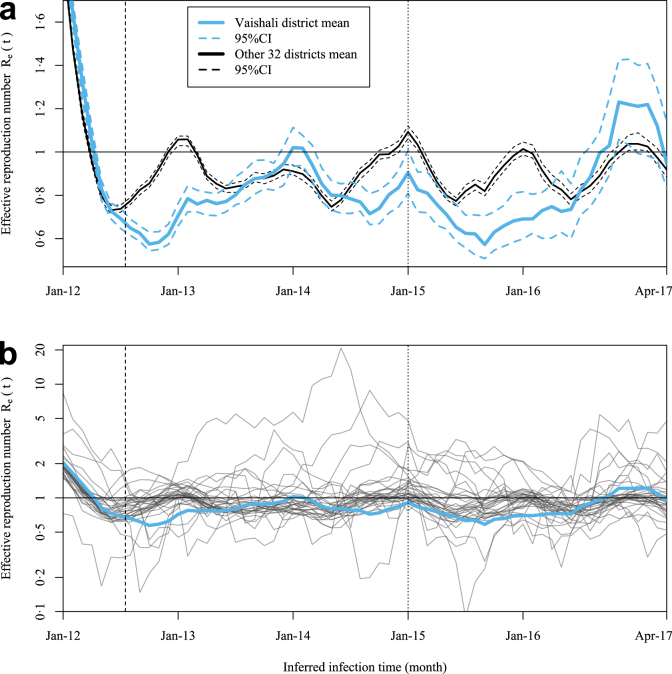
Effective reproduction number ${\overset{ˆ}{R}}_{e}\left(t\right)$ for Vaishali & 32 other districts as means (a) and as 33 separate districts (b). The inferred infection times on the x axes was calculated by subtracting the mean incubation period and mean onset-to-diagnosis time (7-month total shift) from the diagnosis month. The dashed vertical line indicates the length of the 7-month sliding window used to smooth the ${\overset{ˆ}{R}}_{e}$ estimates: before this date estimates are unreliable as they only include partial data within this sliding interval. The dotted vertical line indicates the start of the modelled intervention.

The estimated district-specific effective reproduction numbers ${\overset{ˆ}{R}}_{e}\left(t\right)$ generally follow an annual seasonality (Fig. 3b) which supports using seasonality in the model (Eqns. 2:1 & 2:2). Compared to the average trend of the other 32 districts, Vaishali saw sustained ${\overset{ˆ}{R}}_{e}<1$ during 2012/3, with the second noticeable sustained reduction around the pilot start (Fig. 3a): after summer 2015, Vaishali’s ${\overset{ˆ}{R}}_{e}$ did not return to a seasonal peak around January 2016 unlike the mean of the other 32 districts. However, this effect only lasted the 2015/6 season and Vaishali’s ${\overset{ˆ}{R}}_{e}$ resurged at the end of 2016. Given 2017’s lower incidence, the impact of this above-one ${\overset{ˆ}{R}}_{e}$ in terms of new cases would have been less than if it had occurred at 2015’s case levels.

### 3.4 Pilot model estimation

The final model selected consisted of a Negative Binomial distribution with population offset, annual sinusoid in epidemic & endemic components to account for seasonality, time-specific endemic intercept for high/low incidence, a distributed-lag epidemic component with a single change-of-intercept in Vaishali in January 2015, a constant distributed-lag contribution from directly-adjacent districts in the neighbourhood component, fixed intercept means in the epidemic component (one for Vaishali & one for the other 32 districts), and overdispersion by high/low-endemicity districts (Eqns. 1 & 2, SI §S6–9 & Fig. S4). The final model fitted better than the base model (ΔAIC=−308·3) and showed a reasonable fit to the observed case counts for Vaishali (SI Fig. S5) but across all districts was prone to overestimating low counts (SI Fig. S6). We chose January 2015 as the pilot start month $\tau^{*}$ as it had the lowest AIC.

Table 1 shows the parameter estimates for the intervention effect which can be interpreted as follows. For Vaishali pre-pilot, an estimated average 67·8% of the weighted sum of the previous 12 months’ case counts contributed towards the current month’s case count, versus 70·8% (95%CI 67·7–73·8%) for other districts. This means a hypothetical same-sized epidemic in any of the other districts would take slightly longer to die out on average than if it was to occur in Vaishali. During the pilot, this 67·8% contribution was estimated to fall (by 27·3%) to 49·3% for January 2015 onwards, where the pilot effect confidence interval represents a significant drop (*i.e.* 8·8–45·8%). The estimated endemic contribution per district since January 2012 (based on a mean district population of 2·8 million) was practically nil (∼0 cases/mo) for low-incidence settings (<11 observed cases/mo), whereas high-incidence settings (≥11 observed cases/mo) are estimated to get 3/6 cases/mo for low/high seasons, respectively. In absolute terms the seasonality term contributed more to the epidemic component than endemic (SI Fig. S5)—0·77 times at the November minimum & 1·31 times at the May maximum of the epidemic component. Each district received an estimated 0·5% average contribution from each of the adjacent districts’ weighted sum of their previous 12 months’ cases. The standard errors of all parameters were within reasonable bounds. Parameters were mostly insensitive to pilot start month τ, however, the pilot effect on the epidemic component $\alpha_{\text{pilot}}^{\left(\lambda \right)}$ and Vaishali-specific intercept $\alpha_{\text{Vaishali}}^{\left(\lambda \right)}$ did differ by up to 13% & 7%, respectively, versus their value for a January 2015 start. The changes in the mean parameter of OD distribution had negligible effect on both RQ1 & RQ2 (SI Table S2).

**Table 1:**
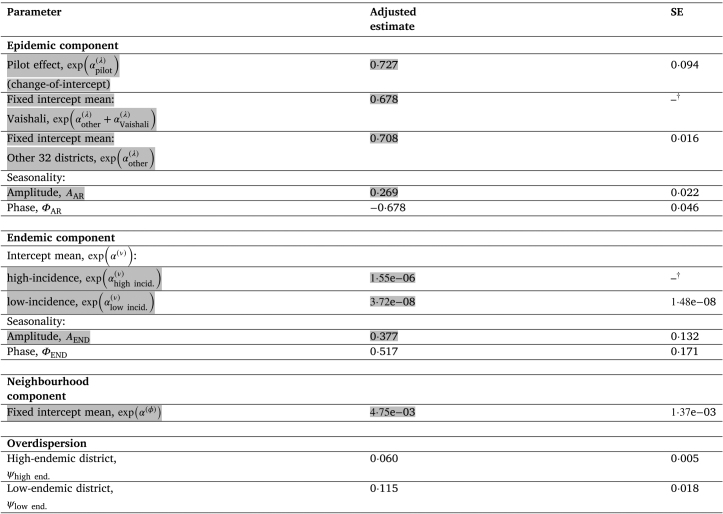
Final model parameter estimates. Those referenced in § 3.4 are  . Mathematical notation explained in Eqns. 1 & 2 in § 2.3. Some parameters (${}^{†}$) are combined from individual ones for interpretability but standard errors are not provided (SI §S9).

The fit of the final model was superior (AIC=10281·7) to the base model (AIC=10590·0) and similar to the counterfactual model (AIC=10286·3). The final & counterfactual models were also better in ranked probability score (RPS=2·50&2·46, respectively) than the base model (RPS=2·81) in prediction for 2015–2017 (p<1e-05 & p<2e-05, respectively, Permutation test).

### 3.5 Estimating cases averted

The counterfactual model showed reasonable predictive performance (SI §S9–10) before the pilot, notwithstanding the last four months of 2014, where the limited duration of this test period caused convergence issues (SI Fig. S7). The final model (SI Fig. S8b) produced forward predictions for 2016–2017 generally sharper & closer to the observed time series than those of the counterfactual model (SI Fig. S8a). Predictions of both models into 2017 were less robust to large departures from the mean trend, *e.g.* the unexpected July 2017 peak in observed cases (SI Fig. S8b), as the epidemic component was diminished by this point. Predictive performance of the final model was poorer at extrema, especially high counts (SI Fig. S10).

**Fig. 4:**
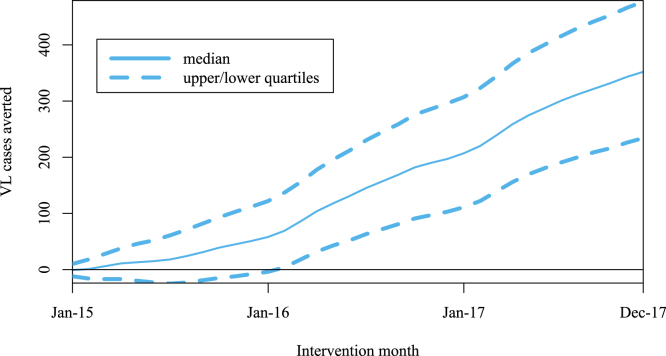
Estimated cumulative cases averted since the pilot start.

Simulations comparing pilot & counterfactual models suggest a median 352 (IQR 234–477) cases were averted in Vaishali during the pilot from January 2015 (2% of 100,000 simulations had estimated negative cases averted), which would have accounted for an estimated 31% of cases if there had been no intensified control (Fig. 4).

Analysing the year-on-year incidence decreases that could have occurred anyway under the counterfactual model, the pilot was estimated to have averted additional cases, as a median percentage of the total cases estimated under the counterfactual model, of 93·9% (IQR 37·5–203·3%) from 2015–2016 and 29·0% (IQR-42·9–137·5%) from 2016–2017 (Fig. 4).

## 4 Discussion

This study comes at a critical point in VL elimination, where high-endemicity districts are predicted to be the hardest in which to reach the elimination target (Le Rutte et al., 2018). Our analysis of the Vaishali pilot study suggests that combining existing interventions with special attention to quality, might contribute to additional reductions in VL incidence.

Descriptive analysis suggests a significant change in the case counts in Vaishali for the first two pilot years 2015–2016 relative to other districts, which is supported by our detailed spatiotemporal analysis that accounts for decreasing trends in cases pre-pilot and neighbouring district effects. When the study started, 15 out of 16 blocks in Vaishali were above the elimination target of 1 case/10,000 people/year, but all blocks apart from Raghopur (where flooding interrupted the pilot in August 2017) are now below the target. Model simulations characterising the pilot period suggest that several hundred cases have been averted since 2015, which was robust to changes in the OD distribution.

We cannot conclusively attribute the additional decline in case counts in Vaishali from 2015 to the intensified control programme because this is an observational study. For internal validity of an ITSA, the continuity assumption must be met so that one is reasonably confident that “no other interventions or confounding covariates than the treatment of interest in analyses changed” at the intervention start month (Bärnighausen et al., 2017). As the pilot & initial decline were concurrent and because no other widespread interventions were in place (SI §S1), we conclude that the additional decline was most likely due to the intensified interventions.

### 4.1 Limitations

This study does not apportion how much each of the pilot’s triad of interventions contributed to the decline nor does it include covariates that describe the time-varying susceptibility of sandflies to the deployed insecticides. Modelling suggests this pilot’s high 90% household coverage per block (SI §S2:2) would have been insufficient alone to reach disease elimination (Fortunato et al., 2021). In addition, a recent study in two highly-endemic districts of Bihar suggests IRS, as implemented under the national control programme, has a negligible impact on sandfly abundance (Poché et al., 2018). Vector abundance, insecticide susceptibility & IRS coverage data from Bihar’s districts would allow further investigation.

‘Single-world’ matching of counterfactual simulations to their corresponding pilot simulations could produce a similar point estimate for cases averted but with lower stochastic variation (Kaminsky et al., 2019), producing the averted estimate with a narrower uncertainty band (*c.f.* Fig. 4) but is beyond this study’s scope. A control group is also lacking as the 32 comparison districts could have unobserved confounders distributed heterogeneously across them, which limits the external validity of the analysis; in addition to inferences coming from a single district.

We do not know the treatment information of some Vaishali cases that chose nearby district hospitals nor other districts’ cases migrating into Vaishali, which could affect the estimated contributions of the epidemic & neighbourhood terms in the model to Vaishali’s case counts. It is also unclear how drug supply may have impacted incidence since the national programme introduced single-dose liposomal amphotericin B in 2015–2016. Some of the largest differences among the 32 non-Vaishali districts are the VL endemicity & mean OD (Jervis et al., 2017), however, our model does not account for these heterogeneities. If ASHA training reduced onset-to-diagnosis (OD) times, and thus infectious durations & subsequent incidence, this would have also shortened the DD distribution, meaning that our inferences & prediction are biased. However, we expect any large reductions in the infectious duration would only marginally affect the OD distribution as the mean infectious period was only 11% of the mean DD.

Underreporting of cases, estimated at 15%–18% in Vaishali in 2012–2013, with a non-uniform age distribution, may have affected our results (Das et al., 2016, Jervis et al., 2017). However, NVBDCP introduced mandatory VL reporting state-wide for the public sector on 7 January 2016. Although HIV/TB-VL coinfection data is included in the monthly cases, we have been unable to stratify their status in the model due to this data only being available since 2015. In a Vaishali district hospital in 2011–2013, VL admissions who were unknowingly HIV+, had OD times on average 3 weeks longer (Burza et al., 2014a); their underdiagnosed HIV-VL status accounted for 2·4% of admissions, rising to 5% in middle-aged men. This may also be important if HIV-VL-coinfected individuals contribute disproportionately to transmission (Burza et al., 2014b). If they do, then the pilot effect in 2017 for Vaishali, a district with a rising proportion of HIV-VL coinfections, may be underestimated. Furthermore, PKDL cases are not incorporated into the analysis as case counts were unavailable from 2012 but recent studies suggest they contribute significantly to transmission as VL incidence declines (Mondal et al., 2019, Chapman et al., 2020).

Despite these limitations, we recommend further pilots in highly-endemic settings with additional collection of time-varying district covariates and assessment of cost effectiveness. Commendably, the WHO roadmap now has three 2030 targets (WHO, 2020a, WHO, 2020b):

1. PKDL elimination (all PKDL cases detected & treated from recovered VL cases followed up for 3 years)
2. VL case fatality rate (<1% nationally)
3. reaffirming the VL elimination target (all blocks in India at <1 new/relapsing VL case per 10,000 population).

Thus widening research questions to these recent goals would support India’s efforts towards VL elimination.

## 5 Conclusion

Can intensified control reduce VL incidence more quickly in a highly-endemic district? Our robust analysis shows that observed VL case counts did fall more quickly in Vaishali district than other districts, in line with previous crude analyses (Kumar et al., 2017, Kumar et al., 2020) and estimates an additional outcome indicator as ‘cases averted’. Since the design of this study (2014), VL policy now covers PKDL burden & VL mortality. We believe there is justification for piloting our approach in other highly-endemic settings, contingent on improvements in study design & analysis (§ 4.1 & SI §S13), to meet these policy advances.

## CRediT authorship contribution statement

**Vijay Kumar:** Pilot conception & design, Secondary data collection, Writing - original draft, Supplementary information. **Niyamat A. Siddiqui:** Secondary data collection, Data analysis & interpretation, Writing - original draft, Literature search, Supplementary information, Critical article revision. **Timothy M. Pollington:** Analysis conception & design, Data analysis & interpretation, Writing - original draft, Literature search, Figs. & tables, Supplementary information, Critical article revision. **Rakesh Mandal:** Pilot conception & design, Pilot implementation, Data analysis & interpretation, Writing - original draft, Literature search, Supplementary information, Critical article revision. **Sushmita Das:** Secondary data collection, Pilot supervision & resources, Writing - original draft, Critical article revision. **Shreekant Kesari:** Pilot conception & design, Pilot implementation, Writing - original draft. **Vidyanand R. Das:** Pilot supervision & resources, Critical article revision. **Krishna Pandey:** Pilot supervision & resources, Writing - original draft, Critical article revision. **T. Déirdre Hollingsworth:** Analysis conception & design, Data analysis & interpretation, Analysis supervision & resources, Writing - original draft, Literature search, Figs. & tables, Critical article revision. **Lloyd A.C. Chapman:** Analysis conception & design, Data analysis & interpretation, Analysis supervision & resources, Writing - original draft, Literature search, Figs. & tables, Supplementary information, Critical article revision. **Pradeep Das:** Pilot conception & design, Pilot supervision & resources, Writing - original draft, Critical article revision.

## Declaration of Competing Interest

The authors declare the following financial interests/personal relationships which may be considered as potential competing interests: VK, NAS, SK, VNRD, KP & PD were the permanent employees of RMRIMS. RM was a Ph.D. student under its Dept. of Vector Biology. They initiated this institutional study on the instruction of the Directorate General of Health Services, Ministry of Health & Family Welfare, Govt. of India. PD had full access to the data and final responsibility for publication submission. TDH, LACC & TMP have no conflicts of interest.

## Appendix A Supplementary data

The following is the Supplementary material related to this article. We detail the study design, analysis & modelling in SI §S1–14 (DOI: 10.5281/zenodo.5579140) and provide a *policy-relevant items for reporting models in epidemiology of neglected tropical diseases* (PRIME-NTD) summary (SI Table S3), to assist in communicating “the quality & relevance of modelling to stakeholders” (Behrend et al., 2020). Analysis code is available at github.com/t-pollington/ITSA.

MMC S1 .
