# Supplementary material for: Impact of intensified control on visceral leishmaniasis in a highly-endemic district of Bihar, India: an interrupted time series analysis
Source: Epidemics. 2022 Jun;39:100562. doi: 10.1016/j.epidem.2022.100562 (PMC9188270; doi:10.1016/j.epidem.2022.100562)
Supplement: MMC S1 — . [file mmc1.pdf]

Supplementary information (SI)

Contents

Pilot description 1
S1. Visceral Leishmaniasis (VL) control under the national programme . . . 1
1) Routine control activities . . . 1
2) Management hierarchy . . . 2
3) Early case detection & management . . . 2
S2. Intensified control for Vaishali district under this pilot . . . 3
1) Early case detection & management . . . 3
2) Improved IRS . . . 3
3) IEC activities . . . 5

Descriptive analysis methods 5
S3. Year-on-year comparisons of monthly case numbers before/during the pilot . . . 5
S4. Global Moran's I for between-district spatial correlation . . . 5
S5. Effective reproduction number $\hat{R}_e$ time profiles . . . 5

Inputs into the spatiotemporal model 5
S6. Obtaining the diagnosis-to-diagnosis (DD) distribution . . . 5
S7. Framework behind the three components of the base spatiotemporal model . . . 6
S8. Overdispersion cut-off choice . . . 6

Model development 7
S9. Model building . . . 7
S10. Model validation . . . 11

S11. Estimating cases averted 12

S12. Further model developments 12

S13. Source of the data & PRIME-NTD summary 16

Pilot description

We describe in detail the implementation and the context of this regional VL control at the time of the pilot.

S1. Visceral Leishmaniasis (VL) control under the national programme

1) Routine control activities

- Early case detection & management, mostly as passive surveillance followed by annual active case detection with a 'camp approach', which is less sensitive and uses a weak referral system. Liposomal amphotericin B in a single dose of 10mg/kg was the first-line VL treatment, and combination therapy (paromomycin-miltefosine injection for 10 days) was the second-line treatment followed by other regimens, *e.g.* amphotericin B emulsion, miltefosine (28 days) & amphotericin B deoxycholate in multiple doses as per availability; this was also the case for Vaishali district[1] under the pilot study.

[1]Note that throughout the main text, supplementary information & code, all mentions of 'Vaishali' refer to the district within Bihar state, rather than the smaller Vidhan Sabha constituency with the same name, which is within Vaishali district.

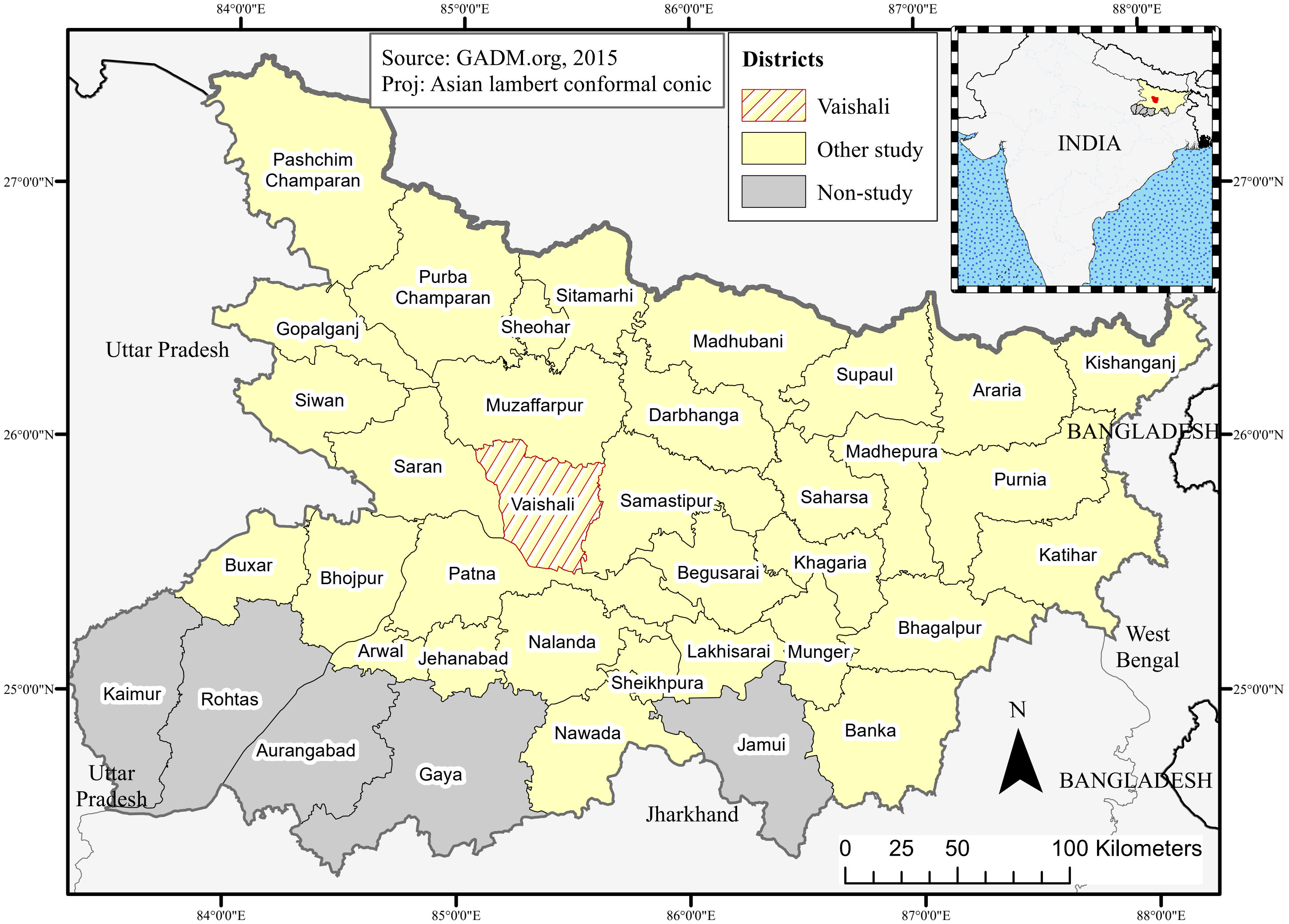

Figure S1: **Study map.** The pilot district of Vaishali is the hashed region on the main map and the red region on the top-right inset map. Shapefile sourced from GADM [GADM, 2015]. Produced in ArcMap™.

- Indoor residual spraying (IRS) using DDT (dichlorodiphenyltrichloroethane) in earlier rounds (50% wettable powder applied at 1g/m^2) and then alpha-cypermethrin (a synthetic pyrethroid: 5% wettable powder at 25mg/m^2) was introduced at different times across Bihar state in 2015 once DDT resistance in sandflies was detected. During the two IRS rounds, insecticide was sprayed in human dwellings & cattle sheds up to 1·8m height. Usually the first IRS round started February–March and then May–June for the second. Village selection was based on passive case reports, *i.e.* any village or hamlet reporting VL in the past 3 years qualified for 100% IRS coverage in that round. Districts of Bihar typically received varying levels of supervised IRS since there was no squad-level supervision, only at a block level.
- Unstandardised information, education & communication (IEC) activities with low coverage.

Neither the pilot nor comparison districts were known to have been supplied insecticide-treated nets by RMRIMS (Rajendra Memorial Research Institute of Medical Sciences), NVBDCP or by others. However, the WHO TDR programme did provide logistical support across Bihar.

2) Management hierarchy

At the district level, IRS was monitored by one District Vector Borne Disease Control Officer & one District Vector Borne Disease Consultant. However, at the block (Public Health Centre or PHC, subdistrict)-level, IRS activity was managed by one Kala-azar Technical Supervisor, with at most one roving camp for active case detection (ACD) at any time.

3) Early case detection & management

There had previously been accredited social health activist (ASHA) training in Bihar since 2012. A Grand Challenges Canada®-funded project in March–April 2012 & October–December 2013, conducted ASHA training in Paroo (Muzaffarpur

district) & Marhoura (Saran district) blocks; whereas Sahebganj (Muzaffarpur) & Baniyapur (Saran) blocks received single training in October–December 2013 [Das et al., 2016b]; but as the training was not implemented comprehensively in these districts, it has not been included in the model herein. From these four blocks approximately 1,000 ASHAs were trained in groups of 100–150 by RMRIMS in VL/post–kala-azar dermal leishmaniasis (PKDL) identification, transmission, treatment & IRS [Das et al., 2016b]. In 2014 the following districts' blocks also received two rounds of ASHA training ending in September 2014: Muzaffarpur (1/16), Saran (1/20), Siwan (1/19), Khagaria (1/7), Saharsa (7/10) & Vaishali (1/16) (blocks trained/total in parentheses); the single Vaishali block of Raghopur received two more rounds of ASHA training in September 2014 as part of this pilot study's intensified intervention for all 16 Vaishali blocks as described in §S2 [Das et al., 2016a, Government of India., 2015].

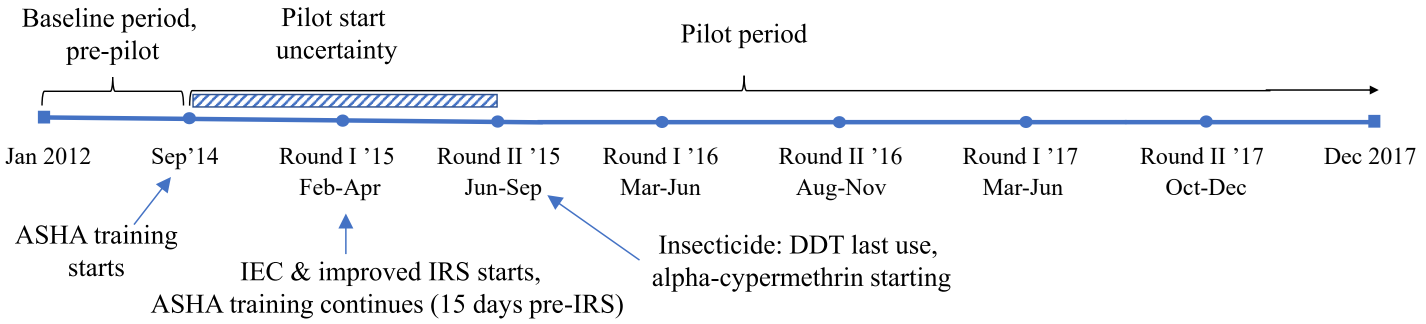

Figure S2: Annotations indicate the start months of the intensified control elements and circular dots mark the biannual ASHA training, IEC & IRS training rounds. The hatched bar marks the period of pilot scale-up where it was uncertain when interventions had reached full strength.

S2. Intensified control for Vaishali district under this pilot

Vaishali is composed of 16 blocks with their own VL control programme supervisor. Additionally, under intensified control, block-level supervisors were selected by and originated from RMRIMS based in Patna, or from their respective block. However, all spraying squads were recruited from each block. Similarly, insecticide & pump equipment were delivered through the District Vector Borne Disease Control Office, Vaishali to blocks and then to villages—in 2015 RMRIMS provided equipment in 7 Vaishali blocks out of 16 but from 2016 it was given to all blocks by the District Vector Borne Disease Control Office. Vaishali district had a total population of 3·50 million in 2011 [Government of India., 2015] and this pilot covered all of its 1,569 villages. RMRIMS staff supervised the pilot, which was composed of three elements:

1) Early case detection & management

The case referral system was strengthened through ASHA training in a variety of ACD approaches. A total of 2,431 new & existing ASHAs across all 16 blocks received two training rounds between 21–29 September 2014. Each trained ASHA worked exclusively on VL & PKDL case detection covering 200 households and was linked by name to the village's microplan; so they were separate from other ASHAs in Vaishali who were outside of this study and monitored other diseases. This training programme was repeated 15 days before each IRS round during the study. Complicated cases of VL were referred to the Samrat Ashoka Tropical Disease Research Centre Hospital (RMRIMS), Patna, Bihar.

The particular ACD approach used was context dependent: house-to-house screening (blanket approach) in villages with five or more VL cases; or for newly-detected VL villages the index case approach—where 50 m surrounding a newly-detected VL case is actively surveilled throughout the year. Furthermore when high incidence was recorded in a focal area, then temporary mobile roving teams (camp approach) would intervene using four camps over an interval of 3–4 months, starting on the same months as IRS (Table S1). In the absence of cases, the standard passive surveillance was followed.

2) Improved IRS

In Vaishali district the IRS insecticides, their concentration, mode of application & village selection were identical to the national programme (§S1) apart from the additions detailed here.

To conduct IRS activities in Vaishali, 24 block supervisors were selected & trained (as per WHO IRS monitoring & supervision criteria [WHO, 2010]) by RMRIMS and assigned to blocks. For IRS monitoring, monitors were also selected & trained by RMRIMS and assigned to each squad for each block. All block supervisors had their own motorbike with daily fuel provision from RMRIMS. Monitors were selected from the locality for spraying, whereas squads were still recruited from the national IRS programme at the district-level by the Vector Borne Disease Control Office (VBDCO), Hajipur. Insecticide,

spray pumps & other equipment were delivered through the VBDCO to blocks and then to villages. The total IRS coverage during the study was 1,145 villages, however this varied between rounds as villages' endemic status changed. IRS coverages by round were as follows: 1,144 villages (Round I 2015); 1,078 (Round II 2015); 995 (Round I 2016); 1,001 (Round II 2016); 1,046 (Round I 2017); 1,067 (Round II 2017).

Initially, supervised IRS with DDT was conducted at 90% household coverage within a block. Later, alpha-cypermethrin was introduced with quality checks, as earlier insecticide sensitivity tests had found DDT resistance. During the DDT era the usual reason for not reaching full coverage was refusal or locked households, as the residents were away working in their fields [Kumar et al., 2009]. Urban/peri-urban properties had higher refusal rates as people with lower socioeconomic status were worried about the effect on their retail goods, while people with a higher socioeconomic status did not think that it affected them or they had protection from living in concrete-walled properties. This indicates how supervision is key to IRS coverage.

**Date**	**IRS round**	**Notes**
February–April 2015	I	DDT<br>IQK for 8 blocks<br>All districts: use stirrup pump except Vaishali where<br>7 blocks use compression pumps (RMRIMS-obtained)<br>& 9 use stirrup. 1 squad/monitor
June–September	II	Saran/Muzaffarpur/Vaishali: DDT for 1st 15 days then SP<br>Other districts: DDT
March–June 2016	I	SP<br>Compression pump (LSTM-obtained) used in all districts<br>2 squads/monitor
August–November	II	
March–June 2017	I	
October–December	II	

Table S1: **IRS schedule for Vaishali district.** DDT = dichlorodiphenyltrichloroethane; IQK = insecticide quantification kit; SP = synthetic pyrethroid.

A summary of the IRS schedule is described in Table S1. Two IRS rounds were conducted annually with the help of 166 spray squads. DDT was used in the first round of 2015 for all districts and in the second only for the first 15 days in Saran, Vaishali & Muzaffarpur districts then alpha-cypermethrin thereafter. All districts received alpha-cypermethrin from 2016 onwards. In Vaishali district, IRS was performed using stirrup & compression pumps. In the first & second rounds of 2015, seven blocks used the compression pump while nine continued with the stirrup pump. From the first round of 2016 onwards, all 16 blocks used 'Hudson® X-Pert® Sprayer' compression pumps. To monitor the quality & coverage of the spraying activity there were 166 monitors; in 2015 one monitor was assigned to each squad whereas in 2016 a monitor covered two squads. A programme supervisor at the block-level oversaw the work of the monitors.

Each squad comprised six members: five Field Workers & Senior Field Worker. In the teams with stirrup pumps, two pairs operated a pump each, one person mixed the insecticide, and the Senior Field Worker maintained the register & marked stencils onto the entrance of the sprayed house. Spraying with the compression pump enabled three people to spray with three pumps, while a pair made the solution and another acted as the Senior Field Worker.

Village selection in Vaishali district used GIS-based mapping of endemic & non-endemic villages for VL trends & hotspot analysis to prepare the microplans. In addition to endemic villages of the last three years, periphery villages of hotspot villages within 500 m of the endemic village boundary that had had a case in the previous year were also included [Mandal et al., 2018]. This algorithm provided a list of villages for the spray team. NVBDCP decided the start of the first round according to the timing of the first seasonal surge in *P. argentipes* sandfly densities. The start of the second round was often dictated by the end of the first round plus a gap of twelve weeks, and by access, *e.g.* if flooding risk from the monsoon rains had diminished; conversely it could not be too late due to people's reluctance to allow spraying before they re-decorated interior walls for October/November festivals.

During the study period the existing stirrup pump was trialled against a compression pump and the latter found to deliver more uniform results [Coleman et al., 2017]. To assess the quality of spraying, four methods were employed:

i) Visual checks by the senior field worker on the same or next day following spraying, and re-spraying as necessary.

ii) Random checks of spraying quality by the squad monitor of 1 in every 10 of the 60–80 households in each village sprayed; they were re-sprayed if the quality was deemed poor on a household basis; if more than 50% of households was poor then the whole village was re-sprayed.

iii) Sandfly density tests (as an indirect measure) were performed in six houses in one village per block (*i.e.* 16 villages × 6 households = 96 households). One of each of three dwelling types was recorded: 'human only', 'human + cattle in same

dwelling' or 'human only + adjacent cowshed'. Samples were taken during a single night at 15 days before IRS, and 2, 4 & 12 weeks afterwards. Sandfly densities by *P. papatasi*, *Sergentomyia spp.* or *P. argentipes* were later determined.

iv) IQKs (insecticide quantification kits) were used to take 3,000 household samples, each from four interior walls of one sleeping room per sampled household at 1·8m, 1·1m & 0·3m height, from eight districts during the first round of 2015. Previous research has shown the IQK's performance as comparable to High Performance Liquid Chromatography—the gold standard [Ismail et al., 2016].

3) IEC activities

Advice on how to prepare for the forthcoming IRS was first conducted during 19–21 February 2015 using audio broadcasts from auto rickshaws. It was conducted at 1,196 locations including marketplaces, private hospitals, block- & panchayat-level health centres, rural childcare centres (anganwadis), schools, state & central governmental offices, and households covered down to the ward-level. Supporting literature over the course of the study covered banners (block-level $n = 44$; village-level $n = 495$), hoardings (marketplaces & government offices $n = 52$), posters ($n = 47{,}840$), leaflets ($n = 95{,}680$) & stickers ($n = 47{,}840$). These activities continued 2–3 days before every spray round.

Descriptive analysis methods

These crude analyses provided essential information prior to spatiotemporal model development.

S3. Year-on-year comparisons of monthly incidence before/during the pilot

Ranked analyses were used as some differences are not visually discernible in time-series graphs that plot multiple districts. Incidences were computed by dividing monthly case numbers by the interpolated district population for that month. For each month, the year-on-year comparison of incidence was computed, then a rank was given to the districts according to which had: a) the highest incidence (highest = 1st rank) & b) the largest negative percentage change in its incidence (largest negative change = 1st rank). Comparisons before & during the pilot were then made by taking the rounded mean rank of each district for each period.

S4. Global Moran's I for between-district spatial correlation

Alongside choropleth maps, Global Moran's I statistic was used to assess global spatial correlation in case numbers, for which a positive value ($I > 0$) indicates clustering of similar case numbers; a negative value indicates clustering dissimilarity in case numbers; and a zero value indicates no correlation and served as the null for the subsequent hypothesis test.

S5. Effective reproduction number $\hat{R}_e$ time profiles

The `epiEstim` `R` package was used to estimate the effective reproduction number [Cori et al., 2013, Cori et al., 2020] $\hat{R}_e(t)$ using an estimated diagnosis-to-diagnosis (DD) distribution (§S7). To smooth the estimate a 6-month sliding window was used that matched the mean DD. As the time of the estimate was still in the diagnosis time domain it was shifted by a fixed single 7-month lag (mean of incubation period + onset-to-diagnosis distribution) to show the results in the infection time domain.

Inputs into the spatiotemporal model

The spatiotemporal model structure is parametric in the Negative Binomial & seasonality distributions used, and regional endemicity levels (§S9). However it was also informed by previous epidemiological parameters in the disease-specific diagnosis-to-diagnosis distribution (§S8).

S6. Obtaining the diagnosis-to-diagnosis (DD) distribution

The sum of normalised weights of 12-month lagged data accounts for the *diagnosis-to-diagnosis (DD)* distribution for VL, D_T, which was estimated as {0·08 [1st lag at $t-1$], 0·11, 0·13, 0·13, 0·12, 0·10, 0·09, 0·07, 0·06, 0·05, 0·04, 0·03 [12th lag at $t-12$]}. This is simulated from:

i) previous incubation period (IP) model estimate from a Bangladesh study using a zero-truncated Negative Binomial distribution with shape parameter = 3, probability of success = 0·35 [Chapman et al., 2015, Chapman et al., 2020] & maximum time of 24 months, which produces a sample distribution with mean of 6 months.

ii) onset-to-diagnosis (OD) time estimate [Jervis et al., 2017] from Bihar in 2012–2013 using a Lognormal distribution with log mean = 3·5, log standard deviation = 0·7 & maximum time 281 days, that produces a sample distribution with mean 1·3 months. We assumed that diagnosis & treatment occurred at the same time as field studies suggest they are only separated by 1–2 days anyway[Jervis et al., 2017].

From these simulated distributions the diagnosis-to-diagnosis distribution, D_T, can be generated (Eqn. S1). Note that the OD distribution was randomly drawn twice for the independent time intervals of case 2 (infectee) & case 1 (infector). By truncating the DD below one month & above 12 months, only ∼9·7% of DD intervals are missed from this range.

$$D_T = \text{Diagnosis-to-diagnosis interval}_{1\to 2} \sim \text{IP}_{\text{case 2}} + \text{OD}_{\text{case 2}} - {}^1\!/\!{}_2\text{OD}_{\text{case 1}}. \tag{S1}$$

S7. Framework behind the three components of the base spatiotemporal model

There is a process producing the observed cases $Y_{i,t}$ in any district i for a certain (diagnosis) month t given the cases seen in the previous 12 months $Y_{i,t-1}$, (for a lag-12 autoregression). This process is assumed to follow a Negative Binomial distribution with conditional mean $\mu_{i,t}$ & variance $\sigma^2_{i,t}$ (Eqn. S2).

$$\mu_{i,t} = e_{i,t}\nu_{i,t} + \lambda_i \sum_{T=1}^{12} D_T Y_{i,t-T} + \phi_i \sum_{j\neq i}\left(\omega_{ji}\sum_{T=1}^{12} D_T Y_{i,t-T}\right)$$

$$\begin{aligned}
\text{where: } & \ln(\nu_{i,t}) = \alpha^{(\nu)}_{i,t} && \text{(endemic component)},\\
& \ln(\lambda_i) = \alpha^{(\lambda)}_{\text{other}} + \mathbb{1}_{\{i=\text{Vaishali}\}}\alpha^{(\lambda)}_{\text{Vaishali}} && \text{(epidemic component)},\\
& \ln(\phi_i) = \alpha^{(\phi)}_{i} && \text{(neighbourhood component)},\\
& D_T = \text{diagnosis-to-diagnosis interval distribution weightings (normalised).}
\end{aligned}$$

Equation S2: **Base model.**

The cases observed in the previous 12 months cannot fully account for those observed in the current month because of noise in the temporal correlation of a district's cases with itself or its immediate (first-order) neighbours. In using this framework [Held et al., 2005] we assume there is a directly-observed process of autoregressive effects from the same district or its neighbours (epidemic & neighbourhood components, respectively) and an indirectly-observed process of background transmission (endemic component) from unobserved symptomatic or asymptomatic individuals. These three components sum to give the conditional mean $\mu_{i,t}$ and the full process we observe (Fig. S3). The directly-observed process can be inferred from spatiotemporally-*local* information of the case numbers of the last 12 months in the district and its immediate neighbours; whereas the indirectly-observed process is inferred by fitting to the pre-specified time-varying membership of the high/low-incidence stratum across Bihar state, to estimate a district-averaged monthly contribution.

Unpacking the base model (Eqn. S2). Negative Binomial distribution with three components:

'endemic' ($e_{i,t}\nu_{i,t}$): cases in the same district caused by a time-specific background unobserved transmission term $\alpha^{(\nu)}_{i,t} \in \left\{\alpha^{(\nu)}_{\text{low incid.}}, \alpha^{(\nu)}_{\text{high incid.}}\right\}$ that takes a lower or higher value according to whether district i in month t has fewer than 11 cases, or 11 or more, respectively. A population offset $e_{i,t}$ accounts for the higher case numbers expected in districts with larger populations.

'epidemic' $\left(\lambda_i \sum_{T=1}^{12} D_T Y_{i,t-T}\right)$: cases correlated with a weighted sum of the last 12 months' cases in the same district i. This component was represented by two fixed intercepts: one for other districts $\alpha^{(\lambda)}_{\text{other}}$ and one for Vaishali, $\left(\alpha^{(\lambda)}_{\text{other}} + \alpha^{(\lambda)}_{\text{Vaishali}}\right)$. The offset term was not included here since the study's cases occur in a minority subgroup of the district's population, so are assumed to be dependent on the cases arising in the epidemic process rather than the wider population.

'neighbourhood' $\left(\phi_i \sum_{j\neq i}\left(\omega_{ji}\sum_{T=1}^{12} D_T Y_{i,t-T}\right)\right)$: recent case importation from adjacent districts j ($\omega_{ji} = 1$ if i & j are adjacent, else 0).

District differences are encapsulated as control effectiveness, which includes the pilot effect in Vaishali later in Eqn. S3 (epidemic component), two high/low endemicity levels (endemic component), and strength of transportation links & human flow between districts (neighbourhood component).

S8. Overdispersion cut-off choice

The Negative Binomial distribution produces non-negative predictions and can account for overdispersion arising from increased variability due to unobserved covariates or time-aggregated incidence [Held, 2020]. As the endemicity distribution of

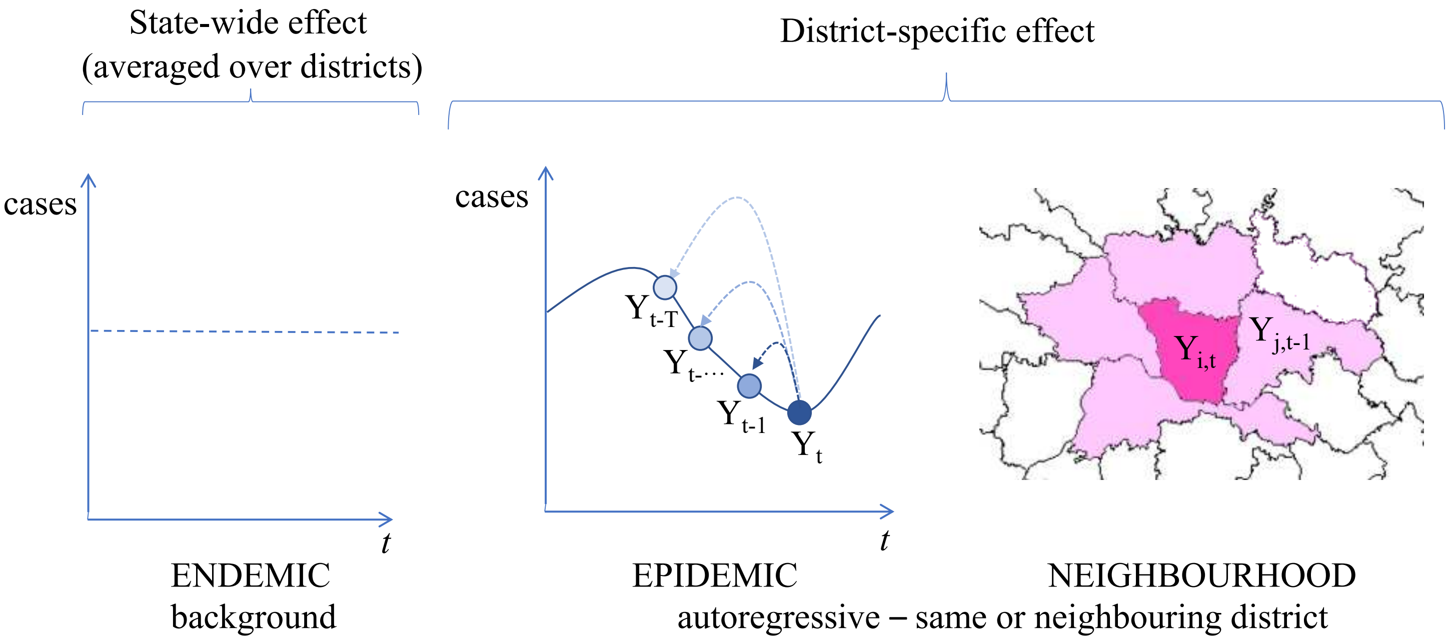

Figure S3: **Model composition.** The respective three components of the Held et al. spatiotemporal model framework [Held et al., 2005].

districts (histogram of districts' total cases during the study) resembles a two-component mixture model (Fig. S4), overdispersion was dichotomised into $\psi_{\text{high end.}}, \psi_{\text{low end.}}$, for high & low endemicity districts, respectively, at a cutoff of 1,000 total cases during the study. This parsimonious approach was preferred rather than introducing a separate overdispersion parameter (ψ_i) for every district.

Model development

S9. Model building

Each fit used all the districts' data for the whole time series (unless indicated) to ensure a good fit to Vaishali's neighbours. Sometimes we extracted Vaishali-specific information from the output for further calculation or graphs. Parameters were inferred using an iterative scheme built into the `surveillance` & `hhh4addon` packages[2] that minimised the model's likelihood using the `nlminb` optimisation method in the `hhh4_lag()` function [Paul and Held, 2011]. Starting from the base model (Eqn. S2) the model fit was sequential to reach the final (pilot) model (Eqn. S3). We evaluated the relative contributions of the time-varying components through a plot of the fitted components alongside the observed cases (Fig. S5).

Model development can be summarised as follows:

Pilot model = Base model + . . .

pilot effect: impact was modelled via a single step change in the intercept in the epidemic component for Vaishali only, to capture changes in the time series of cases when the pilot began (Eqn. S3: epidemic component). This answered the first research question (*Has intensified control additionally contributed to the decline in VL cases in Vaishali, vs. other districts?* (§1.4)).

pilot start month: due to the uncertainty of when each of the three control elements may have started to have an impact on diagnosed cases, we treated them as having a single combined effect, assumed on January 2015 (Fig. S2). Thirteen possible pilot start months τ (September 2014–September 2015 inclusive) were tested and the best model with τ^* chosen that minimised the AIC. To assess the sensitivity of the pilot model parameters to the start time we also report their range when the start month is varied (§3.4). September 2014 was the lower bound as ASHA training had started by then, while September 2015 was the upper bound: if IRS in the first half of 2015 had failed due to DDT resistance, the second round may still have become effective after insecticide change (Fig. S2).

seasonality: annual sinusoid in epidemic and/or endemic components combinations was trialled.

[2] `hhh4addon` is a branched development, additional to `surveillance`

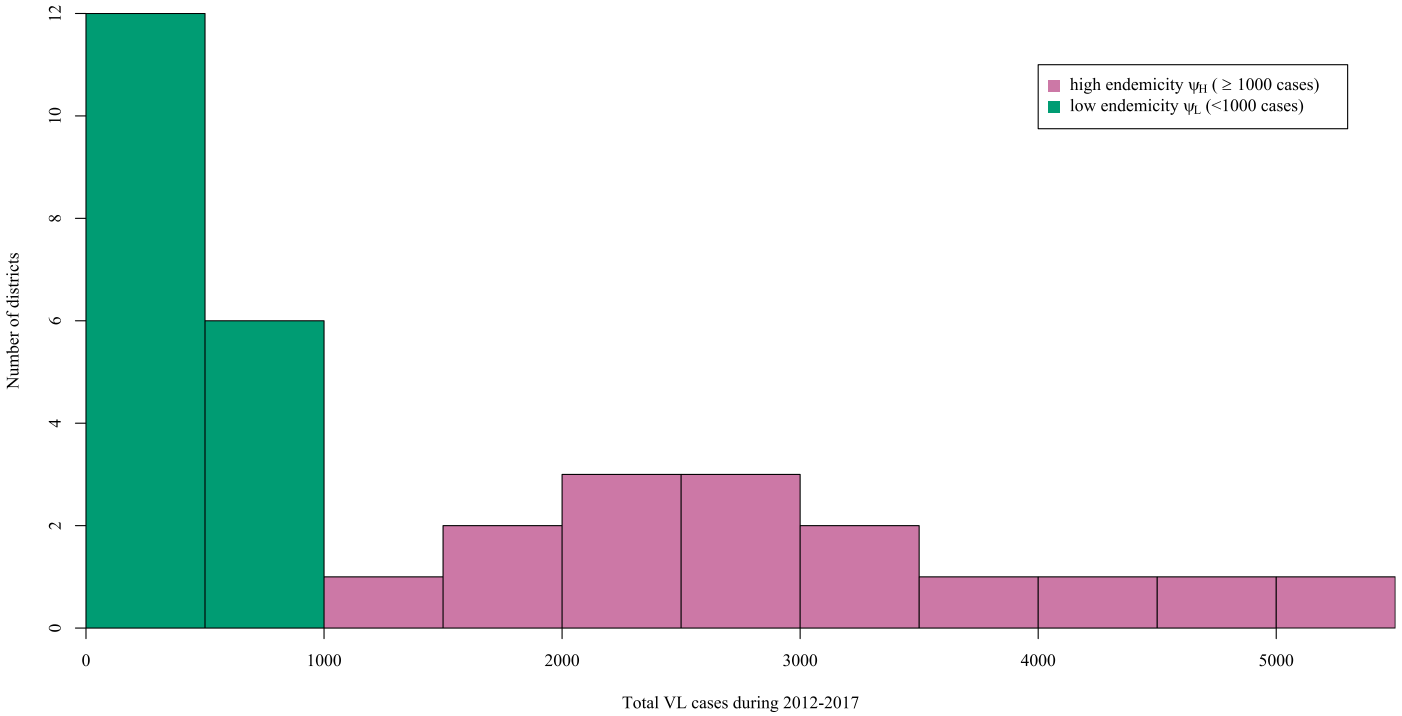

Figure S4: **Overdispersion classification.** District frequency by total VL cases to decide cut-off for high/low overdispersion assignment

The final pilot model is shown in Eqn. S3.

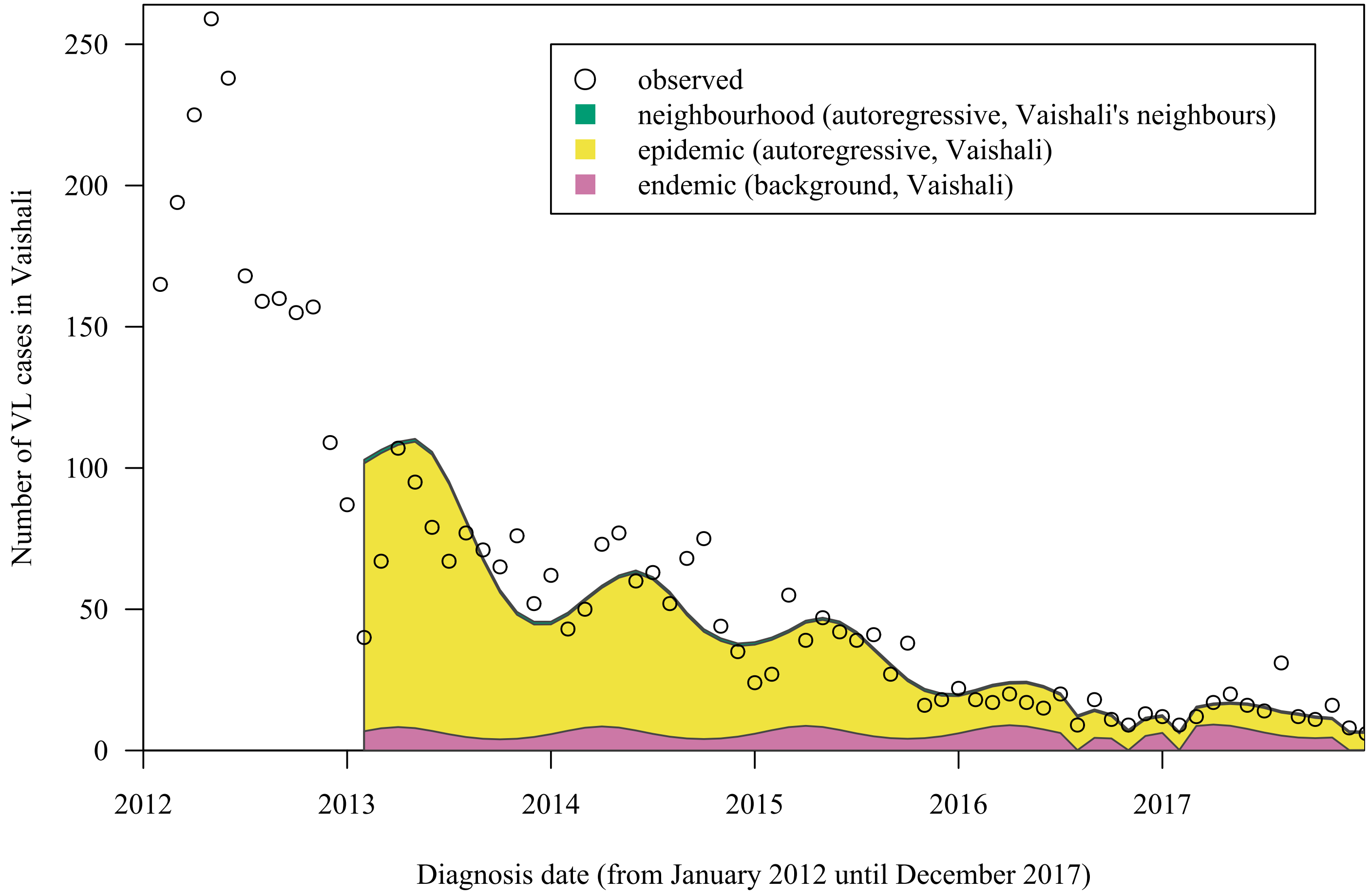

Figure S5: **Model decomposition.** The epidemic component of the model in Vaishali is sandwiched between the neighbourhood & endemic components. After 12 months of lagged data the model component fit starts from January 2013. January 2015 was the chosen pilot start month τ^* for the pilot model, however due to the distributed-lag intervention structure, the reduction in the epidemic component for the first 12 months is gradual via c_t in Eqn. S3. Produced using `surveillance` package in `R` [Meyer et al., 2017, R, 2021].

$Y_{i,t}\big|\{Y_{i,t-12},\ldots,Y_{i,t-1}\} \sim \text{NegBin}(\mu_{i,t},\sigma^2_{i,t})$, for cases $Y_{i,t}$ in district i at month t with conditional mean $\mu_{i,t}$ & variance $\sigma^2_{i,t}$.

$$\mu_{i,t} = e_{i,t}\nu_{i,t} + \lambda_{i,t}\sum_{T=1}^{12} D_T Y_{i,t-T} + \phi_i \sum_{j\neq i}\left(\omega_{ji}\sum_{T=1}^{12} D_T Y_{i,t-T}\right)$$

where t is in month time units, $e_{i,t}$ is the population offset term, $\omega_{ji} = 1$ (if j neighbours i) else 0, with two overdispersion terms $\psi_{\text{high end.}}, \psi_{\text{low end.}} > 0$, $\ s.t.\ \sigma^2_{i,t} = \mu_{i,t}(1+\psi_k\mu_{i,t})$ for $k \in \{\text{high end.}, \text{low end.}\}$ endemicity districts. The three components were formulated as:

$$\ln(\nu_{i,t}) = \alpha^{(\nu)}_{i,t} + A_{\text{END}}\sin\left(\frac{2\pi}{12}t + \Phi_{\text{END}}\right) \qquad \text{(endemic)},$$

$$\ln(\lambda_{i,t}) = \alpha^{(\lambda)}_{\text{other}} + \mathbb{1}_{\{i=\text{Vaishali}\}}\left(\alpha^{(\lambda)}_{\text{Vaishali}} + \mathbb{1}_{\{t\geq\tau\}}c_t\cdot\alpha^{(\lambda)}_{\text{pilot}}\right) + A_{\text{AR}}\sin\left(\frac{2\pi}{12}t + \Phi_{\text{AR}}\right) \qquad \text{(epidemic)},$$

$$\ln(\phi_i) = \alpha^{(\phi)}_i \qquad \text{(neighbourhood)};$$

where $\alpha^{(\nu)}_{i,t} = \alpha^{(\nu)}_{\text{high incid.}}$ if $Y_{i,t} \geq 11$, else $\alpha^{(\nu)}_{\text{low incid.}}$, with τ^* starting pilot month, $A_{(\text{END/AR})}$ annual sinusoid amplitude & phase $\Phi_{(\text{END/AR})}$, fixed intercepts for the 32 districts $\alpha^{(\lambda)}_{\text{other}}$ & Vaishali $\alpha^{(\lambda)}_{\text{Vaishali}}$,

and $c_t = \begin{cases} 0 & \text{if} \quad t < \tau, \\ \frac{\sum_{p=1}^{p=t-\tau+1} Y_{i,t-p}}{\sum_{p=1}^{12} Y_{i,t-p}} & \qquad \tau \leq t < \tau + 11, \\ 1 & \qquad \tau + 12 \leq t. \end{cases}$

Equation S3: **Pilot model.** The counterfactual model was the same but omitted the $\mathbb{1}_{\{t\geq\tau\}}c_t\cdot\alpha^{(\lambda)}_{\text{pilot}}$ term. The c_t term corrects for the first 12 months of the pilot due to delayed effects of the distributed lag.

Selection of the predictive model was based on *predictive performance*. Firstly we compared the predictive performance of the pilot model against the base model to establish that the pilot model was a relative advancement. PIT histograms were also used to check if the pilot model had a constant predictive performance throughout the predicted range [Czado et al., 2009, Meyer et al., 2017].

We then compared the predictive performance of simulations of the (pilot) null model & alternative models with alternative parametric functional forms (linear, Geometric & Poisson) for the diagnosis-to-diagnosis distribution by summing '*one-step-ahead*' sequential model scores [Paul and Held, 2011]. However the DD distribution was shown to have the lowest ranked probability score (RPS) value. The 'one-step-ahead' approach fits up to and including month t and predicts the cases in the next month $t+1$; it uses the difference between observed & predicted cases to form a model score; the model is then refitted with the extra observed data at $t+1$ and the next month's cases predicted, and so on. This approach thus sequentially trials the two models repeatedly, and takes the mean of these scores, then compares the pair's scores. We used the RPS value for comparison, since it gives less weight to extreme departures from the trend of the observed [Abel, 2015]. As the histograms of the distribution of score differences were non-Normal, the non-parametric Permutation Test (using 10,000 simulations) was used to assess the significance of the difference. A p-value less than 0·05 was considered to indicate the alternative model had reasonably better predictive performance. It turned out that the model we attained through AIC selection (pilot model) was also optimal for predictive performance. Therefore the counterfactual model stayed the same as the pilot model, apart from omitting the pilot effect.

Table 1 does not contain standard errors for the combined variables of $\exp\left(\alpha^{(\lambda)}_{\text{other}}+\alpha^{(\lambda)}_{\text{Vaishali}}\right)$ nor separately $\exp\left(\alpha^{(\nu)}_{\text{high incid.}}\right)$, as the `surveillance` package only provides single parameter estimates.

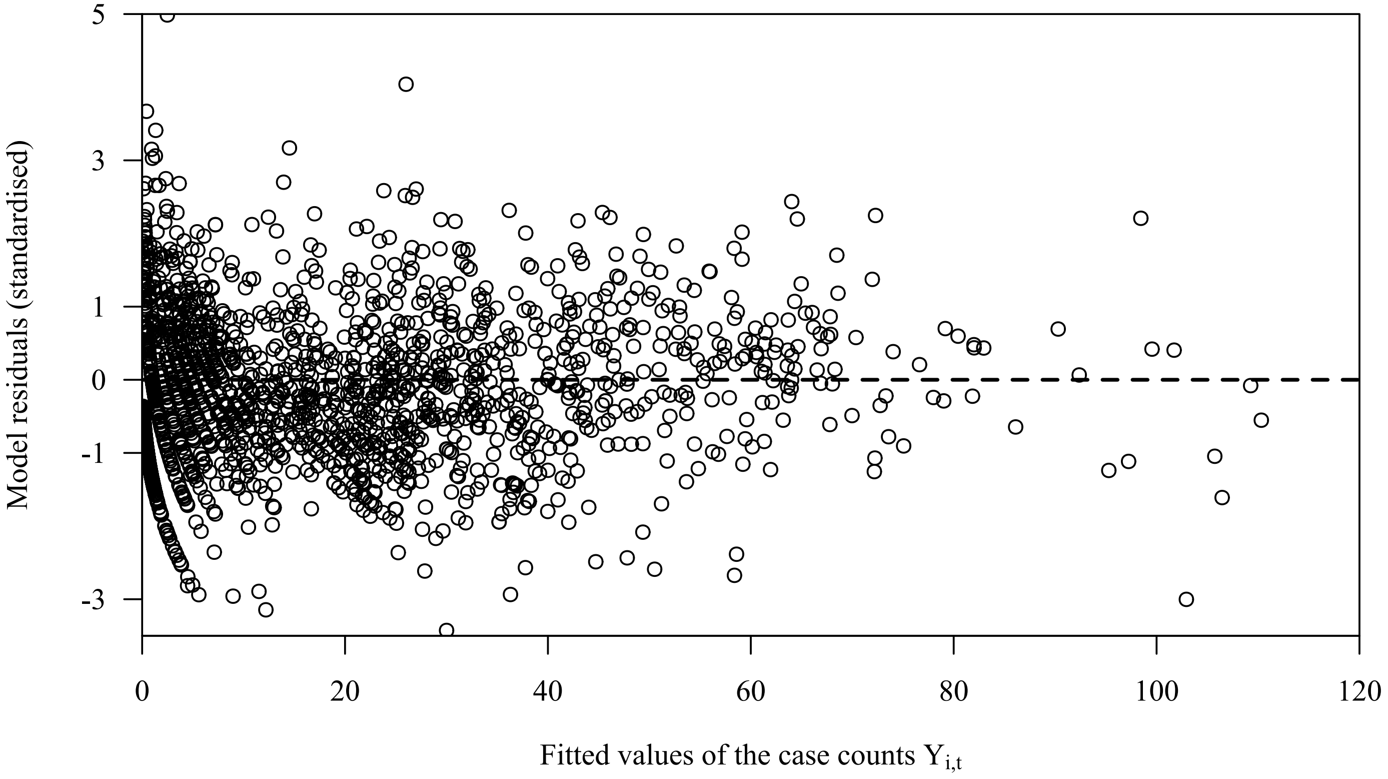

Figure S6: **Pilot model heteroskedasticity when fitted to all 33 districts during January 2013–December 2017.**

S10. Model validation

Heteroskedasticity was present in the pilot model since a higher variance of model residuals was present when the model was fitted to low numbers of cases (Fig. S6). The final counterfactual model made reasonable 'one-step-ahead' sequential forecasts of the monthly case numbers in Vaishali district in 2014 based on a fit to the 2013 data, as assessed visually (Fig. S7), suggesting that the model captured the essential features of the process giving rise to the case counts and could be relied

upon to make counterfactual predictions from 2015, based on the 2013–2014 status quo.

Time series plots were used to visually assess the difference between the pilot & counterfactual models versus the observed case time series with fanplots of the distribution of simulations from sequential 'one-step-ahead' forecasts of January 2016–December 2017 [Abel, 2015] (Fig. S8).

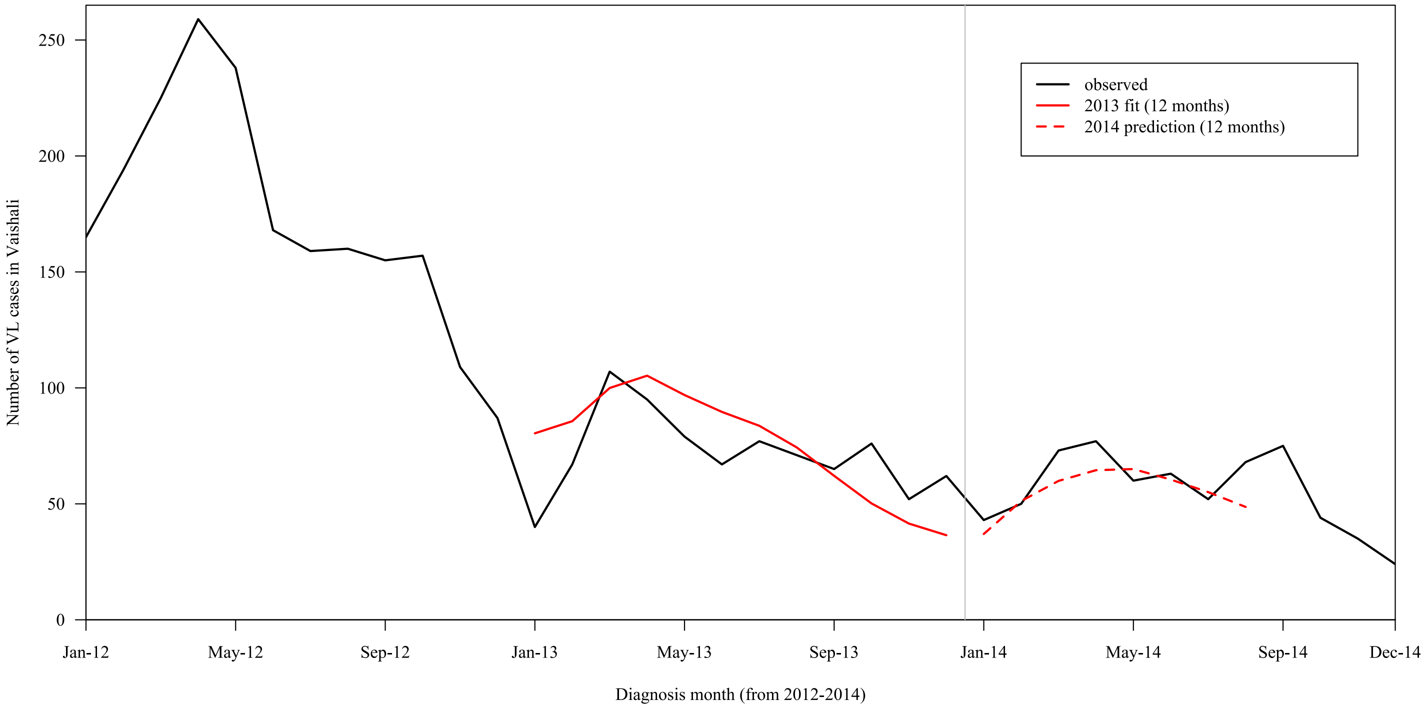

Figure S7: **Counterfactual pre-intervention goodness of fit & predictive performance.** The counterfactual model was fitted to the observed data (black line) during 2013 (on the left-hand side of the vertical grey line) to produce the initial fit (solid red line) for that time series. 'One-step-ahead' forecasts were then sequentially made for progressing months (dashed red line) according to the complete observed time series for the 12 previous months (further details in §S9) [Paul and Held, 2011]. Model convergence problems meant that predictions could not be made for the latter 4 months of 2014—this arose from the limited test dataset and did not appear when using the full dataset.

S11. Estimating cases averted

To answer the second research question *how many VL cases are expected to have been averted by the pilot?* (§1.4), we took the difference in monthly 'one-step-ahead' January 2015–December 2017 forecasts for pairs of: i) simulated cases from the counterfactual model fitted to all data from January 2013–December 2014, initialised with the Mersenne-Twister random number generator seed; & ii) simulated cases from the pilot model. The paired difference (ii−i) was summed for all forecast months to obtain the estimated case numbers averted. We simulated 100,000 pairs to produce an estimated median & IQR for the cases averted.

S12. Further model developments

Introducing a time-varying overdispersion term $\psi_{i,t}$ to account for districts like Vaishali whose endemicities change over the course of the study would likely improve the model fit (similar to how the endemic component intercept $\alpha_{i,t}^{(\nu)}$ is time-specific in Eqn. S2); as would weighting neighbourhood adjacency (ω_{ji} in Eqn. S2) by the proportion of the shared edge to the perimeter of district i. Unfortunately the former is not possible under the current version of the `surveillance` package.

The selected island of 33 districts could underestimate the full neighbourhood effects for two reasons. Firstly, the five unsurveilled districts in south-west Bihar may have had unreported cases. Secondly, the effect of neighbouring states like Uttar Pradesh, Jharkhand & West Bengal or the Nepalese border, which albeit are relatively low-endemicity zones, is not accounted for [NVBDCP, 2017, WHO, 2015]. The latter could be addressed within the `surveillance` framework by modelling entire neighbouring states as additional 'district' units.

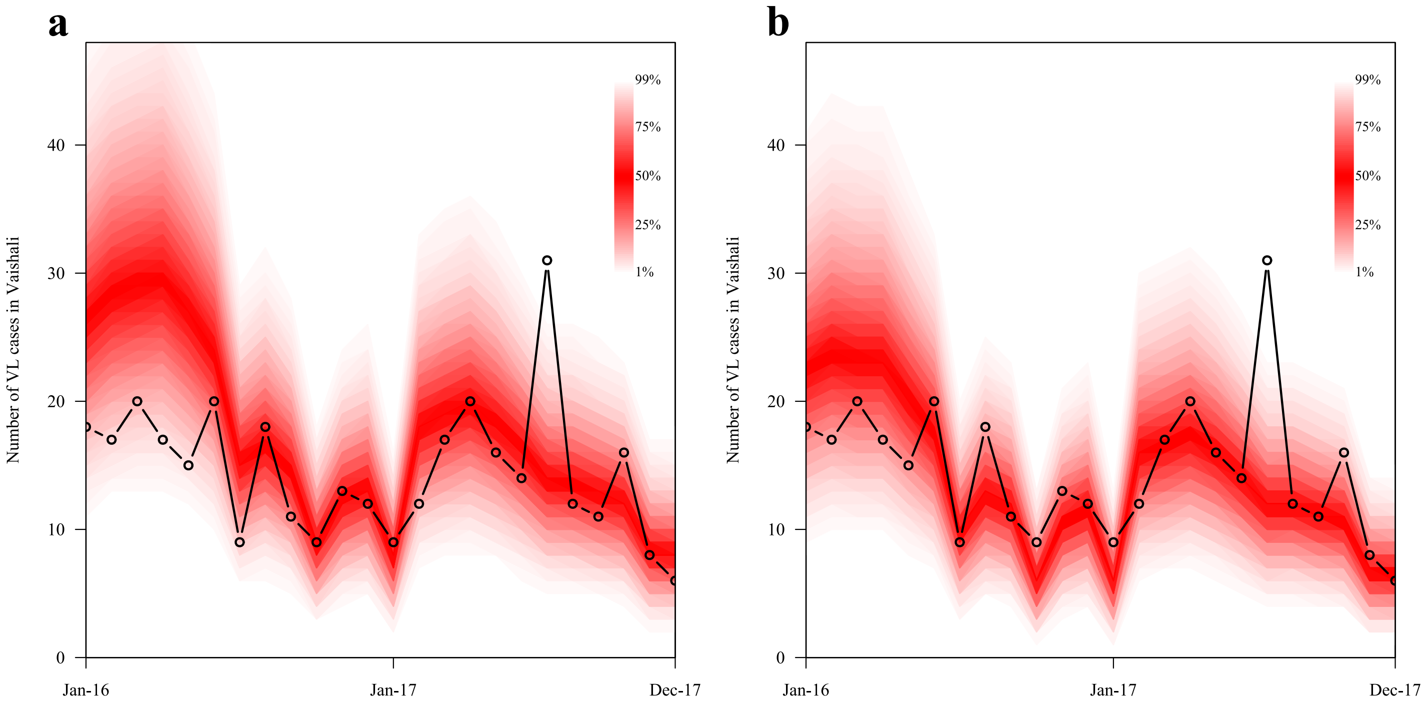

Figure S8: **Fanplots.** Sequential probability distributions for **a)** counterfactual model & **b)** pilot model. The connected black line represents observed cases and the red gradient band indicates sample quantiles about each month's predicted values. Produced using the `fanplot` package in `R`[Abel, 2015].

Given that the disease has a relatively long & varied incubation period it is reasonable to expect that cases are temporally-related through the months. As we use case diagnosis dates, it is unclear how this correlation may be obscured by changes in the unobserved OD time distribution.

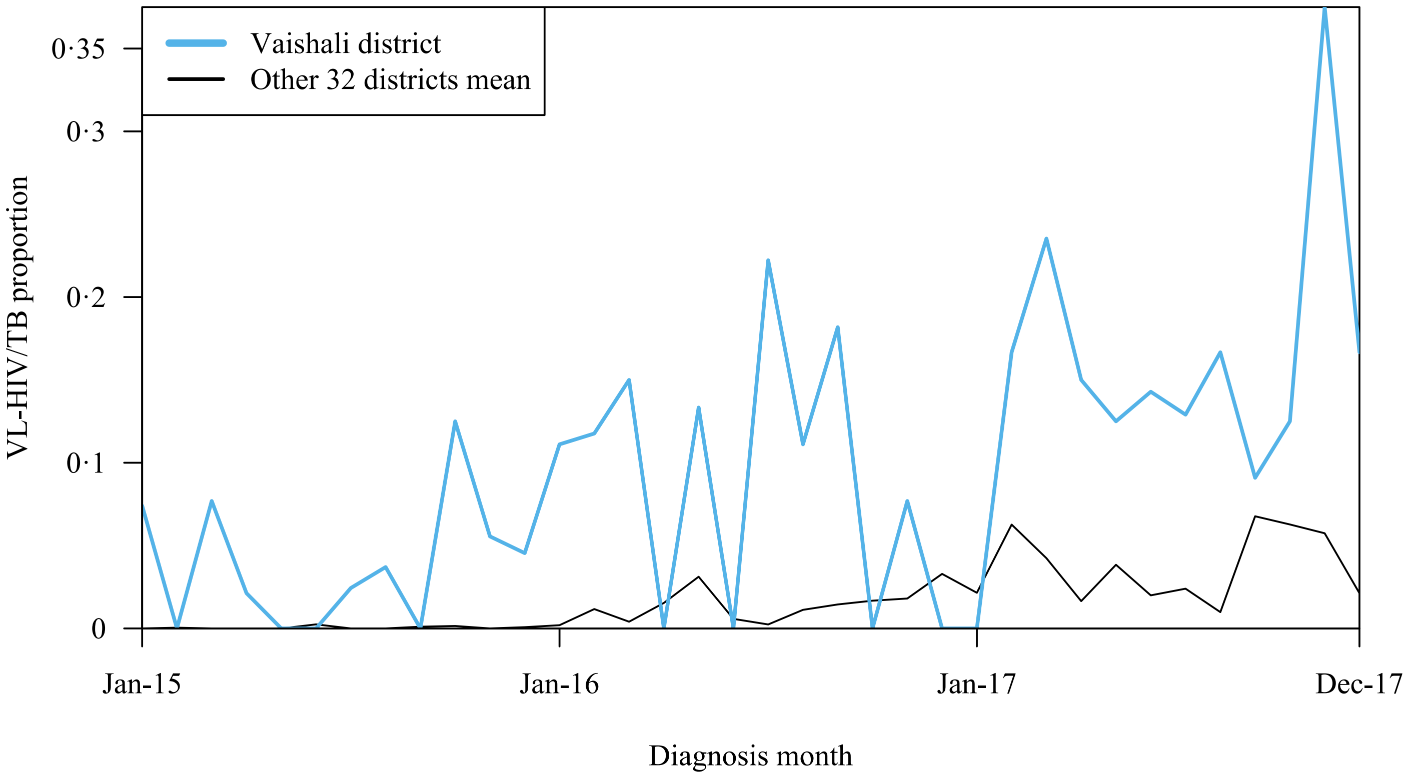

Figure S9: **VL-HIV/TB case proportions.** VL-HIV data from 2015–2017 and additional VL-HIV/TB data since 2017.

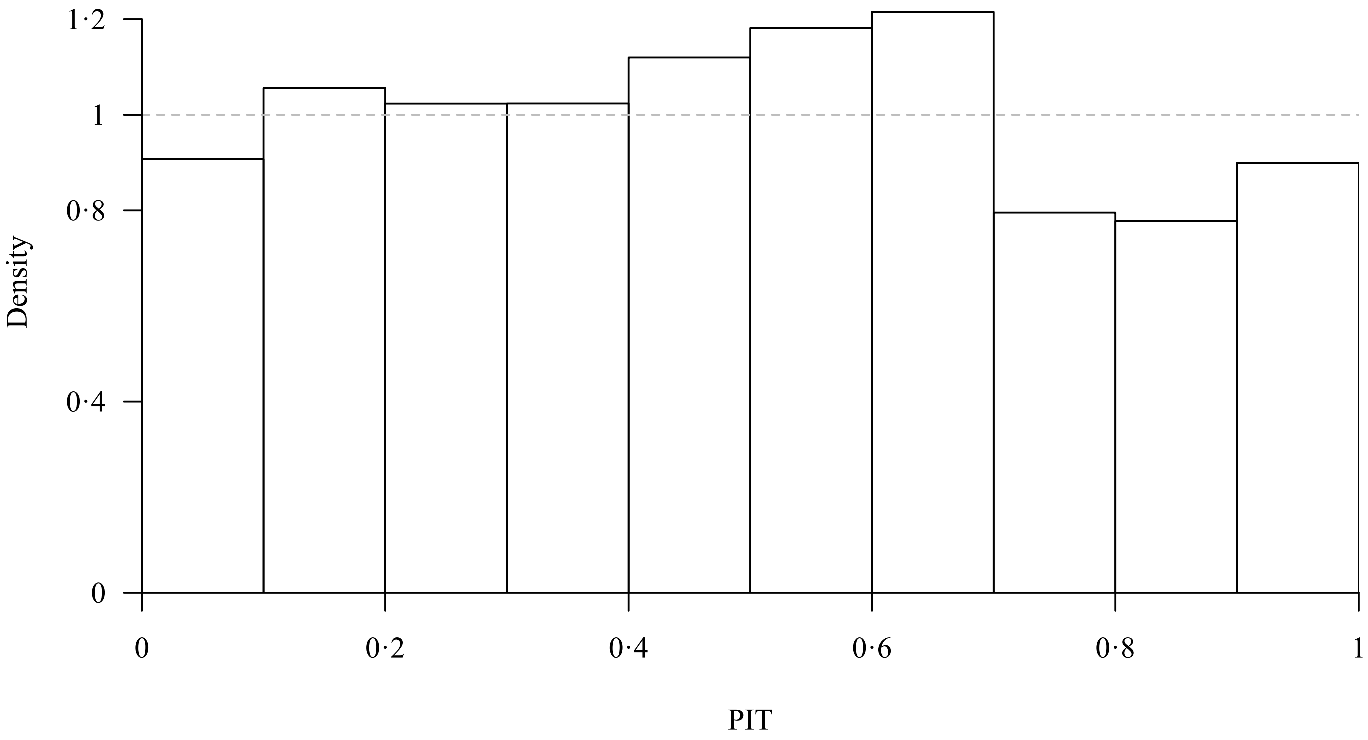

Figure S10: **Probability integral transform (PIT) histogram for the pilot model.** The upside-down ‘U’ shape indicates overdispersion, therefore model predictions at extreme counts will be less reliable.

S13. Source of the data & PRIME-NTD summary

This routine surveillance data originated from the Kala-azar Notification Registry as part of the National Public Health Reporting System maintained by the Office of the Additional Director-cum-State Programme Officer, NVBDCP (Patna). The raw data was inputted electronically and checked for completeness, consistency & data entry errors. Any errors were resolved by the State Programme Office & Nodal Officer of the NVBDCP. The cleaned data was also cross-validated with the NVBDCP's national data repository. This anonymised data aggregated by month & district (admin level 2) was shared with RMRIMS and so was non-personal & non-identifiable since age, sex & village location was not provided. New cases continued to be reported through the usual health system and collated by the NVBDCP; thus this was a secondary data analysis.

Principle	What has been done to satisfy this?	Where is this described?
1. Stakeholder engagement	We plan to share the results of this manuscript at a forthcoming Leishmaniasis conference.	n/a
2. Complete model documentation	The model structure is extensively described both in the main text & supplementary material. Formulae are explicitly given with textual explanation. A compartmental model diagram was not appropriate for this statistical model.<br><br>Analysis code is available at: github.com/t-pollington/ITSA	§2.3–2.4 & SI §S6–11
3. Complete description of data used	Data source & processing described.	§2.1 & SI S13
4. Communicating uncertainty	• Transparency in model assumptions<br>• Model limitations extensively described<br><br>**Presenting hypothesis tests/uncertainties**<br>• Number of Monte Carlo samples used in permutation test provided, as p-value will be conditional on this.<br>• Standard error given for all model parameters. Combined parameters' point estimates provided (*e.g.* $\exp\left(\alpha^{(\lambda)}_{\text{other}} + \alpha^{(\lambda)}_{\text{Vaishali}}\right)$ as it improves results interpretability for the reader)<br>• Intervention effect: cases averted reported with uncertainty (IQR)<br>• CIs in parameter estimates & intervention effect.<br>• IQR of cases averted alongside point estimate (median), so the latter is not interpreted in isolation.<br><br>**Sensitivity analysis**<br>• of model parameters to changes to the intervention start month<br><br>**Simulation uncertainty**<br>• Model comparison in models' ability to predict one-month-ahead was expressed as a p-value.<br>• Fanplots visually expressed simulation uncertainty around predicted values.	SI S9<br>§4.1 & SI S12<br><br><br>SI §S9<br><br><br>Table 1<br><br><br><br><br><br><br>Abstract<br><br><br>§2.3 & 3.4<br><br><br>SI §S9<br><br>SI Fig. S8
5. Testable model outcomes	Code provided shows analysis is reproducible Government data not shareable so reproducibility not demonstrable. Synthetic data unavailable, however the analysis can be run on new datasets with adjacency shapefiles from GADM [GADM, 2015]. Clarity in research questions.	§10<br><br>§1.4

Table S2: How the quality & relevance of modelling is communicated to stakeholders (PRIME-NTD)

References

[Abel, 2015] Abel, G. J. (2015). `fanplot` v4.0.0: An `R` Package for Visualising Sequential Distributions. *R.J.*, 7(2):15–23.

[Chapman et al., 2015] Chapman, L. A. C., Dyson, L., Courtenay, O., Bern, C., Medley, G. F., and Hollingsworth, T. D. (2015). Quantification of the natural history of visceral leishmaniasis and consequences for control. *Parasites & Vectors*, 8(521).

[Chapman et al., 2020] Chapman, L. A. C., Spencer, S. E. F., Pollington, T. M., Jewell, C. P., Mondal, D., Alvar, J., Hollingsworth, T. D., Cameron, M. M., Bern, C., and Medley, G. F. (2020). Inferring transmission trees to guide targeting of interventions against visceral leishmaniasis and post–kala-azar dermal leishmaniasis. *Proc.Natl.Acad.Sci.*

[Coleman et al., 2017] Coleman, M., Foster, G., Deb, R., and Srikantiah, S. (2017). Enhancing surveillance of the visceral leishmaniasis elimination programme in India. In *WorldLEISH6 conference abstracts*, page 1508, Toledo, Spain. worldleish2017.org/documentos/Abstracts_Book_WL6_2017.pdf.

[Cori et al., 2020] Cori, A., Cauchemez, S., Ferguson, N. M., Fraser, C., Dahlqwist, E., Demarsh, P. A., Jombart, T., Kamvar, Z. N., Lessler, J., Li, S., Polonsky, J. A., Stockwin, J., Thompson, R., and van Gaalen, R. (2020). `EpiEstim` v2.2-4: Estimate Time Varying Reproduction Numbers from Epidemic Curves. cran.r-project.org/web/packages/EpiEstim/index.html.

[Cori et al., 2013] Cori, A., Ferguson, N. M., Fraser, C., and Cauchemez, S. (2013). A New Framework and Software to Estimate Time-Varying Reproduction Numbers During Epidemics. *Am.J.Epidemiol.*, 178(9):1505–1512.

[Czado et al., 2009] Czado, C., Gneiting, T., and Held, L. (2009). Predictive Model Assessment for Count Data. *Biom.*, 65(4):1254–1261.

[Das et al., 2016a] Das, P., Matlashewski, G., Das, V. R., and Pandey, R. N. (2016a). *Final GCC Project Report Phase II.* RMRIMS. Patna (unpublished).

[Das et al., 2016b] Das, V. N. R., Pandey, R. N., Kumar, V., Pandey, K., Siddiqui, N. A., Verma, R. B., Matlashewski, G., and Das, P. (2016b). Repeated training of accredited social health activists (ASHAs) for improved detection of visceral leishmaniasis cases in Bihar, India. *Pathog.Glob.Health*, 110(1):33–35.

[GADM, 2015] GADM (2015). Global Administrative Areas: India level 2 shapefiles v2.8. gadm.org, read 2018-02-06.

[Government of India., 2015] Government of India. (2015). Districtwise 2011 census population data. census2011.co.in/data/district, read 2018-10-30.

[Held, 2020] Held, L. (2020). Endemic-epidemic models with discrete-time serial interval distributions for infectious disease prediction talk. In *Advancing Knowledge About Spatial Modeling, Infectious Diseases, Environment & Health Conference hosted by the Fields Institute.*

[Held et al., 2005] Held, L., Höhle, M., and Hofmann, M. (2005). A statistical framework for the analysis of multivariate infectious disease surveillance counts. *Stat.Model.*, 5(3):187–199.

[Ismail et al., 2016] Ismail, H. M., Kumar, V., Singh, R. P., Williams, C., Shivam, P., Ghosh, A., Deb, R., Foster, G. M., Hemingway, J., Coleman, M., Coleman, M., Das, P., and Paine, M. J. I. (2016). Development of a Simple Dipstick Assay for Operational Monitoring of DDT. *PLoS Negl.Trop.Dis.*, 10(1):1–14.

[Jervis et al., 2017] Jervis, S., Chapman, L. A. C., Dwivedi, S., Karthick, M., Das, A., Rutte, E. A. L., Courtenay, O., Medley, G. F., Banerjee, I., Mahapatra, T., Chaudhuri, I., Srikantiah, S., and Hollingsworth, T. D. (2017). Variations in visceral leishmaniasis burden, mortality and the pathway to care within Bihar, India. *Parasites & Vectors*, 10(601):1–17.

[Kumar et al., 2009] Kumar, V., Kesari, S., Dinesh, D. S., Tiwari, A. K., Kumar, A. J., Kumar, R., Singh, V. P., and Das, P. (2009). A report on the indoor residual spraying (IRS) in the control of Phlebotomus argentipes, the vector of visceral leishmaniasis in Bihar (India): an initiative towards total elimination targeting 2015 (Series-1). *J.Vector.Borne.Dis.*, 46(3):225–9. ncbi.nlm.nih.gov/pubmed/19724087.

[Mandal et al., 2018] Mandal, R., Kesari, S., Kumar, V., and Das, P. (2018). Trends in spatio-temporal dynamics of visceral leishmaniasis cases in a highly-endemic focus of Bihar, India: an investigation based on GIS tools. *Parasites & Vectors*, 11(220):1–9.

[Meyer et al., 2017] Meyer, S., Held, L., and Höhle, M. (2017). Spatio-Temporal Analysis of Epidemic Phenomena Using the `R` Package `surveillance` v1.19.1. *J.Stat.Softw.*, 77(11):1–55.

[NVBDCP, 2017] NVBDCP (2017). Kala-azar Cases and Deaths in the Country since 2010. nvbdcp.gov.in/ka-cd.html, read 2018-02-15.

[Paul and Held, 2011] Paul, M. and Held, L. (2011). Predictive assessment of a non-linear random effects model for multivariate time series of infectious disease counts. *Stat.Med.*, 30(10):1118–1136.

[R, 2021] R (2021). `R` v4.1.1: A Language and Environment for Statistical Computing.

[WHO, 2010] WHO (2010). Monitoring and evaluation tool kit for indoor residual spraying. who.int/tdr/publications/documents/irs_toolkit.pdf, read 2018-10-09.

[WHO, 2015] WHO (2015). Status of endemicity of visceral leishmaniasis worldwide, 2015. who.int/leishmaniasis/burden/Status_of_endemicity_of_VL_worldwide_2015_with_imported_cases.pdf, read 2018-02-15.
